# The Ontogeny of the Human Calcaneus: Insights From Morphological and Trabecular Changes During Postnatal Growth

**DOI:** 10.1002/ajpa.70007

**Published:** 2025-02-12

**Authors:** Carla Figus, Kristian J. Carlson, Eugenio Bortolini, Jaap Saers, Francesca Seghi, Rita Sorrentino, Federico Bernardini, Antonino Vazzana, Igor Erjavec, Mario Novak, Claudio Tuniz, Maria Giovanna Belcastro, Jay Stock, Timothy M. Ryan, Stefano Benazzi

**Affiliations:** ^1^ Department of Cultural Heritage University of Bologna Ravenna Italy; ^2^ Department of Integrative Anatomical Sciences, Keck School of Medicine University of Southern California Los Angeles California USA; ^3^ Evolutionary Studies Institute University of the Witwatersrand Johannesburg South Africa; ^4^ Naturalis Biodiversity Center Leiden CR the Netherlands; ^5^ Department of Biological, Geological and Environmental Sciences—Bigea University of Bologna Bologna Italy; ^6^ Department of Humanistic Studies Università Ca’ Foscari Venezia Italy; ^7^ Laboratory for Mineralized Tissue Centre for Translational and Clinical Research Zagreb Croatia; ^8^ Multidisciplinary Laboratory Abdus Salam International Centre for Theoretical Physics Trieste Italy; ^9^ Centre for Applied Bioanthropology Institute for Anthropological Research Zagreb Croatia; ^10^ Department of Archaeology and Heritage, Faculty of Humanities University of Primorska Koper Slovenia; ^11^ Department of Anthropology Western University London Ontario Canada; ^12^ Department of Anthropology Pennsylvania State University University Park Pennsylvania USA

**Keywords:** calcaneal ontogeny, geometric morphometrics, locomotion, trabecular analysis

## Abstract

**Objectives:**

To investigate the developmental changes in the human calcaneal internal and external morphology linked to the acquisition of mature bipedal locomotion.

**Methods:**

Seventy seven micro‐CT scans of modern juvenile calcanei (from perinates to 15 years old) are employed. The chronological period spans from the Middle/Late Neolithic (4800–4500 BCE) to the 20th century. Through a comprehensive approach that comprises geometric morphometric methods and whole‐bone trabecular analysis, the calcaneal growing morphology has been explored.

**Results:**

Morphological changes reflect the development of bipedal locomotion, showing its potential when tracking the major locomotor milestones. The calcaneal shape is immature and almost featureless during the first year of life. The internal architecture is dense and isotropic with numerous thin trabeculae closely packed together. The internal architecture changes to better adapt to variations in load stimulated by a more mature gait by increasing bone mass and alignment, with fewer and thicker struts. The external morphology shows its plasticity by increasing the surface area where greater strain is expected and changing the orientation of the articular facets.

**Conclusions:**

Analysis of morphological changes in the growing calcaneus highlights the importance of an integrative methodology when exploring developmental bone plasticity. The changes in calcaneal internal and external morphologies reflect the different loading patterns experienced during growth, gradually shifting from a more generalized morphology to a more adult‐like one, reflecting major locomotor achievement. Our research shows that although initially genetically driven, calcaneal plasticity may display mechanical influences and provide precious information on tracking the main locomotor milestones.

## 1 Introduction

The evolution of bipedal locomotion fascinates paleoanthropologists and enhances our understanding of human locomotor kinematics (DeSilva et al. 2018; Finestone et al. 2018; Frelat et al. 2017; Harcourt‐Smith 2010; Holowka et al. 2017a, 2017b; Marchi 2015; O'Neill et al. 2015; Ryan and Van Rietbergen 2005; Saers et al. 2016; Shaw and Ryan 2012; Ward 2002). The recent advances in imaging techniques, such as high‐resolution computed tomography (CT) and quantitative three‐dimensional studies, have allowed for more in‐depth and non‐destructive analyses of morphological variation, deepening our knowledge about locomotor behavior in the hominin lineage. In particular, numerous studies have explored how bone functionally adapts to and, to some extent, reflects the behavior of individuals thanks to the mechanism of bone (re)modeling (Barak 2020; Barak, Lieberman, and Hublin 2011; DeMars et al. 2021; Raichlen et al. 2015; Ryan et al. 2018; Shaw and Ryan 2012; Sorrentino et al. 2021; Sorrentino, Stephens, et al. 2020). Modifications in external shape and trabecular bone structure may increase our understanding of locomotor behavior in the past. Trabecular bone, specifically, has proven useful due to its higher remodeling rate than cortical bone, providing a potentially useful tool for interpreting habitual loading in the skeleton (Barak 2020; Barak, Lieberman, and Hublin 2011; Kivell 2016). Bone morphology of recent species, with known behaviors, has proven helpful in inferring behavioral patterns of extinct species. Thus, analysis of trabecular architecture provides additional information that can be combined with internal and external studies of cortical bone morphology, to provide a more complete understanding of how bone reflects habitual loading. Adult morphology is the byproduct of ontogeny, that is, a combination of genetic and epigenetic factors (Carter and Beaupre 2001; Kivell 2016), and mechanical factors (Carter 1987; Carter and Beaupre 2001). Other dynamics such as vascularization, metaphyseal location, and systemic factors (Acquaah et al. 2015; Kivell 2016) have been observed to influence the structural heterogeneity of trabecular and cortical bone in the skeleton (Byers et al. 2000; Cunningham and Black 2009; Fazzalari et al. 1997; Modlesky et al. 2014; Salle et al. 2002). Consequently, developmental patterns should be considered before studying adult morphology.

In the last decades, an increasing number of studies have focused on the development of trabecular bone within different skeletal elements (Colombo et al. 2018; DeMars et al. 2021; Figus et al. 2022; Figus, Sorrentino, et al. 2023; Figus, Stephens, et al. 2023; Gosman and Ketcham 2009; Raichlen et al. 2015; Ryan et al. 2018; Ryan and Krovitz 2006; Saers 2023; Saers, Ryan, and Stock 2020; Saers et al. 2022a, 2022b). In this framework, pedal elements have played a critical role in reconstructing the locomotor repertoires of early hominins.

The foot, the only part of the human body to contact the ground during walking and standing, has been the center of broad research (DeSilva et al. 2019; Fernández et al. 2018; Harper, Ruff, and Sylvester 2021; Holowka and Lieberman 2018; Prang 2015; Sorrentino, Carlson, et al. 2020; Sorrentino, Stephens, et al. 2020; Su and Carlson 2017; Turley and Frost 2014). The modern human gait cycle is a complex set of actions that broadly consists of alternate stance and swing phases during which the foot acts as both a shock absorber and a rigid lever (Griffin et al. 2015). Four main phases can be recognized: (1) initial contact: the knee is relatively extended, and the foot is dorsiflexed, allowing the heel, that is, the calcaneus, to contact the ground. The calcaneus absorbs the shock, and the foot starts to bear the body weight; (2) midstance: the hip is directly above the ankle and the knee is extended. The foot rolls to a flat position, as forces are transmitted from the ground to the lateral side of the foot. The entire body weight is borne by the foot; (3) push‐off: the rigid foot, acting as a lever, passes the forces medially and over the ball of the foot. The plantar flexor muscles contract, pushing the forefoot down to generate a propulsive force generating the push‐off (Hennig and Rosenbaum 1991). The toes flex to grip the ground and finally, this phase ends when the hallux leaves the ground, that is, toe‐off; (4) swing phase: the knee and hip are bent to allow the leg to move forward as the other foot touches down. The heel‐to‐toe and the medial‐to‐lateral transfer of the Center of Pressure (COP) is unique to adult locomotion. The COP is the point of application of the ground reaction forces on the contact area between a person and the substrate, that is, the foot. Its role is fundamental in maintaining stability and balance and preventing falls.

While standing and walking, the body weight is transmitted from the leg to the foot through the talus. From the talus, forces are transmitted posteriorly to the calcaneus and anteriorly to the head of the first and second metatarsals (the “ball of the foot”) and, to a minor extent, to the heads of the other metatarsals. Since the tuberosity of the calcaneus and the heads of the metatarsals are the principal weight‐bearing areas of the foot, the weight of the body is shared almost equally between the hindfoot (calcaneus) and forefoot (metatarsals) (D'Août and Aerts 2008; Moore, Dalley, and Agur 2013, 814). The human foot is characterized by both stiffness and mobility of the midfoot during stance (Holowka and Lieberman 2018). The calcaneocuboid joint has a fundamental role during toe‐off, with the support of the windlass mechanism (Griffin et al. 2015). The cuboid facet as defined by Bojsen‐Møller (1979) resembles an hourglass, “lodged in a little recess of the medioplantar aspect of the calcaneus, where it is held in position by the strong plantar calcaneocuboid ligament, and the flat peripheral part has an inferior extension similar to that on the cuboid.” Asymmetry and convexity of the facet are peculiar characteristics of the human calcaneus (Harper 2023), and are thought to ease the closed‐packed position of the calcaneocuboid joint during inversion, increasing foot stiffness during toe‐off (Bojsen‐Møller 1979; Harper, Ruff, and Sylvester 2021; see also Behling et al. 2023). The cuboid facet is subjected to compressive loads (Giddings et al. 2000). The depth of the cuboid facet allows for increased midfoot flexibility by facilitating inversion/eversion (Holowka et al. 2017a). The calcaneus, that is, the heel bone, is particularly relevant in this context. It helps spread body weight forces while absorbing, also thanks to the presence of the fat‐pad, reaction forces from the terrain during the heel‐strike phase of the mature bipedal gait cycle when its lateral part is typically the first to contact the ground (Rosenbaum and Becker 1997). During walking, the adult human calcaneus is subjected to compressive and tensile forces (Giddings et al. 2000). According to Giddings et al. (2000), the greatest compressive load is found on the posterior talar facet and the calcaneocuboid joint, while the highest tensile forces are experienced by the posterior calcaneal tuberosity, where the Achilles tendon attaches. Bands of trabeculae correspond to these distributions, with compressive trajectories extending posteriorly and anteriorly along the superior half of the calcaneus, and tensile trajectories extending along the inferior half. In contrast, a third system of trabeculae extends along the plantar surface of the calcaneus (Saers, Ryan, and Stock 2020).

Numerous studies have been conducted on calcaneal comparative morphology (Harper, Ruff, and Sylvester 2021), function (Harper 2023; Tsegai et al. 2018), and ontogenetic development in relation to bipedal locomotion (Saers, Ryan, and Stock 2020; Saers et al. 2022b; Zeininger et al. 2018). Saers et al. 2022c demonstrated that age‐related modifications in bone volume fraction are linearly correlated to the changes in locomotor kinetics. These two modifications are, in turn, strongly linked to neuromuscular maturation. Many features of calcaneal morphology have been linked to bipedal locomotion, for example, a robust calcaneal tuberosity, the presence of a prominent lateral plantar process, posterior talar facet curvature, and a deep cuboid facet (DeSilva et al. 2019; Harper, Ruff, and Sylvester 2021, 2022; Holowka and Lieberman 2018; Prang 2015, 2016). Here, we aim to further explore the ontogeny of the human calcaneus by investigating the development of both external and internal morphologies using a whole‐bone approach.

### 1.1 Development of Human Locomotion

During development, and unlike the other tarsal bones, the calcaneus ossifies from two centers of ossification via perichondral and endochondral ossification. It is the only tarsal bone with an epiphysis, even though the talus may have a secondary center of ossification. The ossification of the calcaneus starts in utero and its complete ossification, with the fusion of its secondary center, is completed between 18 and 20 years of age (Cunningham, Scheuer, and Black 2016; Scheuer and Black 2004).

During the first years of life, humans learn how to walk bipedally. This is a fascinating and lengthy process, which has been subject to many studies (Adolph and Franchak 2017; Hallemans et al. 2003, 2006; Mameli et al. 2024; Sutherland 1997; Zeininger et al. 2018). This process is far from linear, however, and is influenced by many factors, as it is strictly linked with socio‐cultural background and different patterns of maturation of musculoskeletal and neurological systems. Different childrearing practices, for example, leg stimulation practices such as massages or passive exercise, as well as neonatal reflexes (Adolph and Franchak 2017; Lacquaniti, Ivanenko, and Zago 2012), may influence the development of locomotor skills. As such, the acquisition of independent bipedal gait in early walkers should be observed with consideration of socio‐cultural background.

For decades, researchers have observed and standardized mostly white and wealthy children within the US middle class, often of European descent (Karasik et al. 2015). This bias has led to standards based on insufficient cultural variation, resulting in infants from different cultures having been classified for decades as precocious or delayed (Lerner, Liben, and Mueller 2015). More recently, considerable cross‐cultural differences have been highlighted (Karasik et al. 2015), and locomotor milestones have been appreciated as varying substantially from infant to infant. Nonetheless, there is a general progression of locomotor development in juvenile humans.

Roughly, infants start to sit independently at around 6 months postnatal, while some infants may begin to crawl as early as around 9 months. Toddlers start to walk on two legs, on average, at around 12 months (Karasik et al. 2012; Sutherland, Cooper, and Daniel 1980), or within a range of 8–18 postnatal months (Adolph and Franchak 2017). These milestones should be considered in a wider context, as they are part of a unique event. The transition from one milestone to another is smooth and they can intermingle for a period (Figus, Stephens, et al. 2023).

Early walkers exhibit a wide base of support, short stride length, and low walking speeds. They do not engage in a heel‐strike or toe‐off pattern, as mature walkers do, and instead contact the ground with flat feet, extended knees, and high hip lift (Hallemans et al. 2006; Sutherland 1997; Zeininger et al. 2018). This pattern is the consequence of a weak tibialis anterior that is unable to dorsiflex the foot during the swing phase, resulting in flat foot contact (FFC) (Zeininger et al. 2018). A flat foot during initial contact offers a wider support base and helps children control their balance (Hallemans et al. 2006), while toe‐off is not developed yet as early walkers are unable to produce enough torque at the ankle to move onward into the swing (Hallemans et al. 2006). In addition, early walkers exhibit substantial step‐to‐step variation, that is, high variation in step length and width, during initial locomotor practice, while they try to maintain balance, which is known to increase metabolic costs during walking (O'Connor, Xu, and Kuo 2012).

In the early developmental phase, at touch down, the foot has a plantigrade position, that is, flat foot, offering a wider support base and effectively increasing stability. As the heel, midfoot, and metatarsals simultaneously touch the ground, these areas experience similar peak pressures. After some months of practice, the lateral‐to‐medial transfer of the COP develops, and early walkers begin to engage in initial plantarflexion, that is, develop a toe‐off and heel‐strike pattern. This pattern develops into initial heel contact (IHC), where peak pressure occurs under the heel at touchdown. The roll‐off phase gradually matures with subsequent practice, and load‐bearing shifts distally to the lateral part of the foot, increasing pressure on the lateral forefoot region (Hallemans et al. 2003). These changes lead to a different distribution in peak pressures under the heel and midfoot, unlike early walkers who distribute pressure more evenly under the entire plantar surface, and foragers walking on naturalistic, uneven forests (Holowka et al. 2022).

According to Bertsch et al. (2004), the adult‐like lateral‐to‐medial transfer of the center of pressure (COP) develops around 18 months of age. An effective heel strike develops around 18–24 months (Sutherland, Cooper, and Daniel 1980; Bertsch et al. 2004). By around 2 years of age, the muscles associated with plantarflexion are adequately strengthened to permit a toe‐off lift, and most children, at this stage, engage in an adult pattern of knee flexion and extension (Zeininger et al. 2018). The walking base is about 70% hip width at the age of 1, about 45% at age 3–4, and reaches adult proportions of around 30% around age 7 (Levine, Richards, and Whittle 2012).

The extended‐hip‐and‐knee‐joint pattern, together with a heel strike and narrow step width, helps use less energy during walking on normal terrains (Holowka et al. 2022) and develops with age as neuromuscular maturation and locomotor kinematics progress.

The longitudinal arch begins to form as soon as walking starts, but it does not fully develop until the age of 4–6 (Bertsch et al. 2004). By age 4, maturation of the central nervous system approaches adult levels, after which gait differences between adults and juveniles are largely attributable to allometry (Sutherland 1997). With increasing longitudinal arch height, peak plantar pressures on the fore‐ and hind‐foot increase, and pressure is reduced at midfoot to adult levels between ages 5 and 6 (Bertsch et al. 2004; Zeininger et al. 2018). However, patterns of muscle activation do not fully resemble those of adults until roughly 15 years of age (Sutherland 1997; Sutherland, Cooper, and Daniel 1980).

### 1.2 Hypotheses

This study aims to investigate the ontogeny of the calcaneus by combining geometric morphometrics and trabecular analysis. The main goal is to contribute to the increasing understanding of the developing juvenile skeleton, by adding information on the calcaneal morpho‐functional changes.

External shape:

Hypothesis 1a *Based on previous studies (Harper* 2023 *; Holowka et al*. 2022 *), we tested the possibility that there might be a separation between a more immature calcaneal shape (compact and rather short) and a more mature one (narrow and slender) (H1a) as the calcaneus develops concurrently with heel strike (Harper* 2023 *), in individuals around and older than 3 years of age*.

Hypothesis 1b *We expect to see a profound change in the cuboid facet after 6 years of age, as gait becomes more mature and adult‐like (e.g., more stereotypical loading and more predictable directions of loading), with a shift to a more adult‐like morphology, i.e., from a more convex shape to a more angled and adult‐like one, with an increase in asymmetry and convexity*.

Internal architecture:

Hypothesis 2a *< 1 year of life (i.e., perinatal and 0–1‐year group). We expect to see an isotropic and dense structure, with numerous and closely packed trabeculae as demonstrated by studies on humans (Figus et al*. 2022 *; Figus, Sorrentino, et al*. 2023 *; Figus, Stephens, et al*. 2023 *; Saers, Ryan, and Stock* 2020 *; Saers et al*. 2022b *) and on Japanese macaques (Saers et al*. 2022a *). During the first year of life, we predict an observable reduction in bone volume as the number of trabeculae decreases and the space between them increases. We further expect that the trabeculae will become more anisotropic. We expect a steady increase in trabecular separation as calcaneus size increases throughout development*.

Hypothesis 2b *1.1–3‐year group. After 1 year of age, when children begin to walk independently, we predict that bone volume will increase, and trabeculae will become thicker and more anisotropic. As trabeculae become thicker, the spacing between them will decrease (Colombo et al*. 2018 *; Figus et al*. 2022 *; Milovanovic et al*. 2017 *; Raichlen et al*. 2015 *; Ryan and Krovitz* 2006 *; Saers, Ryan, and Stock* 2020 *). Transient forces experienced during heel strike may lead to an increase in BV/TV values in the entire posterior part of the calcaneus. These forces may be attenuated during development, to some extent, by the compact aspect of the calcaneus, the heel pad, and the cartilage anlagen (Cunningham, Scheuer, and Black* *2016* *). However, fractures at the apophyseal plate are not uncommon and suggest the presence of great shear strain at this location (Ogden et al*. 2004 *). BV/TV is expected to further increase in the inferior part of the calcaneus after the development of toe‐off when tensile forces act on the plantar ligament*.

Hypothesis 2c *3.1–6‐year group. The neuromuscular system matures, gait kinematics gradually change, including a reduction in step width, and increased body size and motor control. The combination of these changes results in more stereotypical loading from increasingly more predictable directions. These changes should lead to an increase in the degree of anisotropy along with increases in BV/TV through the thickening of trabeculae and a reduction in the average distance between them (Raichlen et al*. 2015 *). Changes in trabecular structure after the age of 6 should be primarily predictable from allometry, as spacing increases with body size (Saers, Ryan, and Stock* 2019 *)*.

## 2 Material and Methods

### 2.1 Sample

This study explores postnatal development of the calcaneus in a sample consisting of 77 modern juveniles aged between birth and 15 years postnatal (Table S1). Non‐pathological left calcanei with minimal or no damage were selected. The right one was selected and virtually mirrored when the left calcaneus was missing. The sample spans multiple sites.

*Osteological collection*: 28 individuals (from the Emilia‐Romagna region: 21 from Bologna, one from Parma, and one from Faenza; and from the Sardinia region: two from Cagliari, three from Sassari) with associated age‐at‐death, sex, and cause of death information (Belcastro et al. 2017, 2022) serve as the control sample. They are part of the Documented Human Osteological Collections (DHOC) of the University of Bologna, consisting of 19th–20th Century individuals from Italy. The selected individuals died from acute diseases. The absence of chronic pathologies is a fundamental prerequisite for inclusion in the sample since a prolonged period of locomotor difficulties could have affected the development of locomotion.

*Archaeological sample*: Age‐at‐death in the archaeological sample was estimated based on the development and eruption patterns of deciduous and permanent teeth (Moorrees, Fanning, and Hunt 1963a, 1963b; Smith 1991; Ubelaker 1987). When no teeth were available, diaphyseal length (Stloukal and Hanáková 1978) and epiphyseal fusion were used for age estimation (Black and Scheuer 1996; Ferembach 1980; Schaefer 2008; for more information, see specific references).

- Eleven individuals came from the Imperial Roman site of Velia. The site, originally founded by the Greeks in 540 BC (Morel 2006), is located on the Italian west coast, near Salerno (Campania, Italy). Numerous archaeological campaigns have been held and, during the 2003–2006 campaigns (Fiammenghi 2003), a necropolis with over 330 burials was discovered. This necropolis, which dates to the I and II centuries AD, yielded both cremations and inhumations, with numerous juvenile burials.
- Six individuals were selected from the collection of St. John's (Cambridge, UK, 1230–1511 AD). This sample comes from the medieval cemetery associated with the Hospital of St. John the Evangelist in Cambridge. The individuals lived in an urban area, but they may have been involved in an intensive agricultural subsistence strategy (Saers, Ryan, and Stock 2020). Their diet was rich in carbohydrates, and health status suggested nutritional, physiological, and pathogenic stress, which was common during this period (Cessford 2015). The Hospital of St. John's was a place of hospitality, as intended by the etymology of the Latin word *hospital*. Therefore, the presence of seriously ill people has been excluded as such individuals were not allowed in the Hospital (Orne and Webster 1995).
- Five individuals were selected from the medieval site of Campochiaro Morrione (Italy), dating to the 7th century AD (Belcastro et al. 2007; Gasparini et al. 2022 and references herein). Grave goods indicate different cultural elements were represented in the site, that is, Barbaro‐Germanic, romano‐byzantine, and steppe‐nomadic. One of the main characteristics of this site is the presence of several horsemen, buried with their horses.
- Five individuals were selected from the Late Antique necropolis of Ostra Vetere (Ancona, Italy), dating between the 6th and 7th century AD. The necropolis was in an area previously occupied by buildings dating between the 3rd century B.C. and the 5th century A.D., which overlooked the forensic area. Between the 6th and 7th centuries, these structures were abandoned, and the pavements, flagstones and spoliation pits were used as a burial ground for several burials that formed a veritable cemetery area, exploited from Late Antiquity to the Early Middle Ages (Dall'Aglio, Franceschelli, and Tassinari 2013; Vazzana 2019).
- Seven individuals come from Suasa, an ancient Roman town located in the present‐day municipality of Castelleone di Suasa (Ancona, Italy). Three individuals were retrieved from the northern necropolis, (1st–4th century AD), while the remaining four came from the cemetery area (4th–9th century AD), uncovered in the garden of the Domus dei Coiedii. Both sites were located along the main street of the city, that is, Via del Foro Romano (Bogdani and Giorgi 2008; Giorgi and De Maria 2013; Vazzana 2019).
- Six individuals were from the middle/late Neolithic layers of a site near Beli Manastir, Croatia (4800–4500 BCE) (Los 2020).

To quantify morphological and structural differences in changes in the loading environment, the sample was divided into five groups (Table 1), based on previous literature (Swan et al. adapted; Figus et al. 2022, Figus, Sorrentino, et al. 2023; Figus, Stephens, et al. 2023), as follows:

- Perinates, which includes individuals around the time of birth who were unable to engage in any mixed locomotor behavior except for spontaneous movements of the lower limbs;
- Neonates and infants (0–1 year), which includes infants that were unable to walk independently and those that engaged in mixed locomotor behavior, for example, cruising, crawling, assisted walking;
- Toddlers (1.1–3 years), which includes infants able to walk with an immature toddling gait without assistance, that is, with a wide walking base, small stride length, and low speed. During this phase, the lateral‐to‐medial transfer of the center of pressures, and a proper heel strike develop;
- Early childhood (3.1–6 years), which includes infants in an intermediate phase between immature and mature gait. During this phase, the longitudinal arch develops;
- Late childhood (6.1–10 years), which includes children who achieved mature and adult‐like locomotion.

**TABLE 1:** Age classes.

Age classes (years)	Group name	Individuals per class	Individuals per class and site						Suasa	Velia
			Beli Manastir	Bologna (La Certosa, Faenza, Parma)	Sardinia (Cagliari, Sassari)	Campochiaro Morrione	Saint John, Cambridge	Ostra Vetere		
0	Perinates	6								6
0–1	Neonates and toddlers	4		2						2
1.1–3	Early infancy	17	1	5		1		3	4	4
3.1–6	Late infancy	11		6					2	3
6.1–10	Childhood	18	2	5		2	4	2	1	4
10.1–15	Adolescence	14	3	6	5	2	2			
Total		77	6	24	5	5	6	5	7	19

### 2.2 Data Acquisition and Segmentation

Bones were scanned using different microCT sources (Table 2) and volume datasets were reconstructed as 16‐bit TIFF stacks. ImageJ v.1.52a (Schneider, Rasband, and Eliceiri 2012) was used to inspect scan quality. Individuals with trabecular damage or rarefaction due to pathological or diagenetic causes were excluded from the analyses. Reconstructed volume data were pre‐processed (e.g., crop or resample) in Avizo v. 9.3 (Thermo Fisher Scientific, Waltham). Heavy sediment or mummified soft tissues were removed from volume data in silico using a Wacom board and the Avizo paint‐brush tool in the label field module. Then, three‐dimensional surface models were created from the segmented microCT data. A White Hat filter was applied to some individuals from Velia to improve contrast between bone material and heavy sediment present within internal spaces of the bones. Segmentation of the image data was first performed following the protocol described by Yazdani et al. (2019, 2020).

**TABLE 2:** MicroCT information.

Sample	Facility	Type of scanner	Voxel size (μm)
Bologna	Center for Quantitative Imaging (CQI), Pennsylvania State University, PA (USA)	General Electric vjtomejx L300 nano/microCT	20–38
Beli Manastir	Zagreb	Skyscan 1076 system (Bruker Corp., Kontich, Belgium)	18–29
Velia	The Abdus Salam International Centre for Theoretical Physics, Trieste, Italy	Microfocus X‐ray computed tomography^a^	18–30
	Rizzoli Institute, Bologna, Italy	Skyscan 1072 system (Bruker Corp., Kontich, Belgium).	18
St. John	Cambridge Biotomography Center, University of Cambridge	Nikon XTH 225 ST, Nikon Metrology	47

^a^ System specifically designed in collaboration with Elettra Sincrotrone (Trieste) for the study of paleontological and archeological materials (Tuniz et al. 2013).

The external surfaces of calcanei from Suasa, Campochiaro Morrione, and Ostra Vetere were acquired with an ARTEC Space Spider 3D structured light laser scanner (0.1 mm resolution) at the Department of Cultural Heritage of the University of Bologna (Ravenna).

#### 2.2.1 Geometric Morphometric Analysis

**FIGURE 1:**
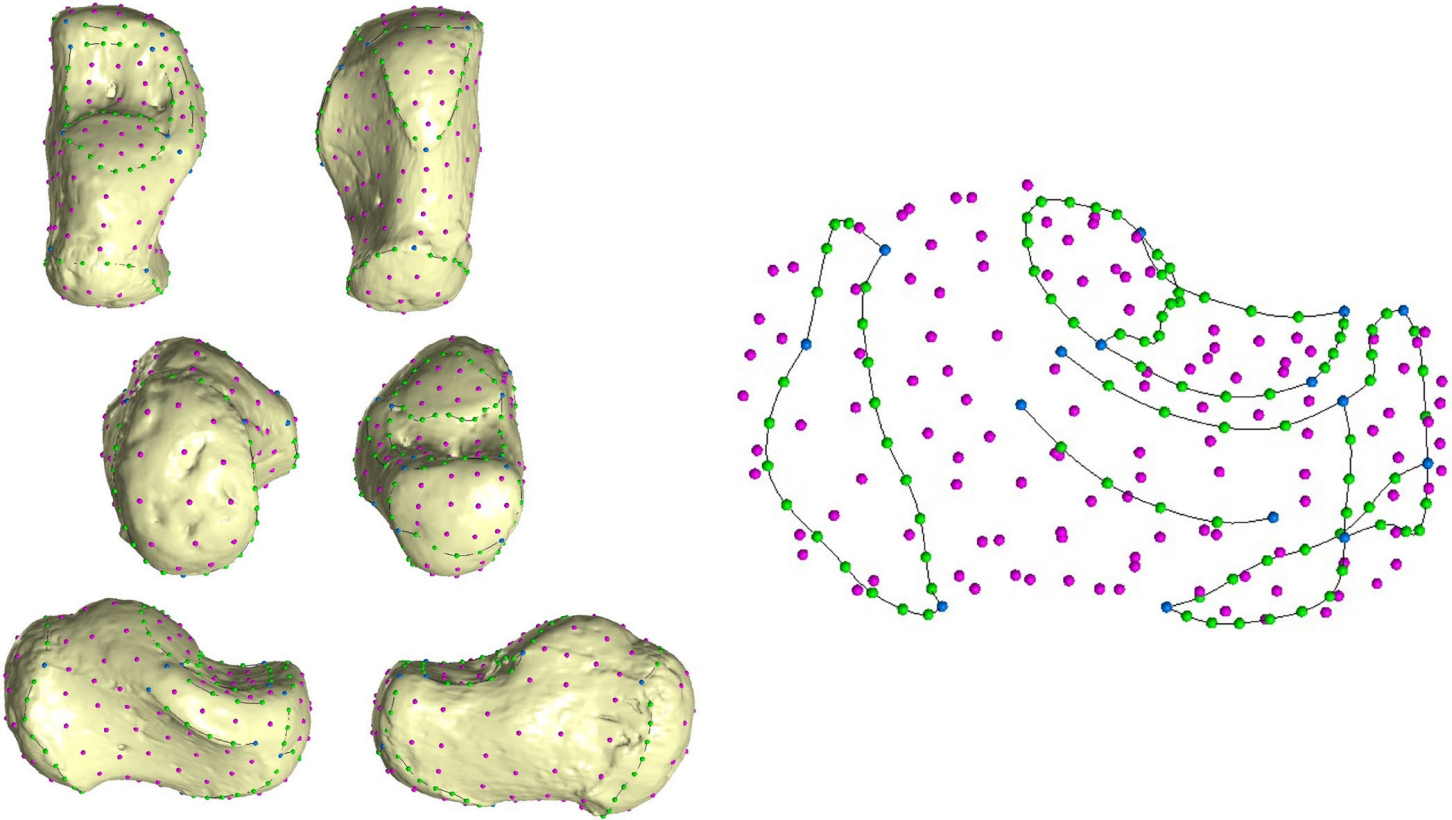
Configuration of (semi)landmarks. Landmarks are represented in blue. Curve semilandmarks are depicted in green, while surface semilandmarks are magenta.

A template of 180 (semi)landmarks (15 anatomical landmarks, 88 curve semilandmarks, and 77 surface semilandmarks, as shown in Figure 1) was created in Viewbox 4 (dHAL Software) on an infant calcaneus (BO‐14‐F, age at death 1 year and 9 months) (Figure 1, Tables 3 and 4). The infant calcaneus has been selected based on its developmental state, that is, it allowed for the use of a configuration of (semi)landmarks compatible with all the targets (see Table S1 for more information about the application of the template on each target).

**TABLE 3:** Configuration of Type III landmarks according to Bookstein (1997).

Description
1—most lateral point of the metaphyseal surface
2—most medial point of the metaphyseal surface
3—most inferior point of the metaphyseal surface
4—most lateral points on the talar articular surface
5—point of contact between the talar articular surface and the sustentaculum tali
6—most proximal point of the sustentaculum tali
7—most proximal point of groove for the Flexor Hallucis Longus
8—most distal point of groove for the Flexor Hallucis Longus
9—point of contact between sustentaculum tali and anterior talar facet
10—most lateral points on the anterior talar facet
11—most inferolateral points on the cuboid facet
12—most inferomedial point on the cuboid facet
13—most prominent point in plantar view
14—most distolateral point of the sinus tarsi
15—most distomedial point of the sinus tarsi

**TABLE 4:** Semilandmarks.

Semilandmarks on curves	*N*
1–2—superior curve of metaphyseal surface	4
1–3—lateral curve of metaphyseal surface	9
3–2—medial curve of metaphyseal surface	9
4–5—inferior curve of superior articular surface	9
4–14—lateral curve of sinus tarsi	4
5–4—superior curve of articular facet	9
5–15—medial curve of sinus tarsi	4
6–9—sustentaculum tali	4
7–8—groove for FHL	4
10–11—lateral curve of anterior talar facet	3
11–12—inferior curve of anterior talar facet	4
12–9—medial curve of anterior talar facet	3
14–15—proximal curve of sinus tarsi	4
Total	88
*Semilandmarks on surface*	N
Metaphyseal surface	14
Lateral surface of the body	24
Superior surface of the body	11
Medial surface of the body	24
Sustentaculum tali	4
Total	77

The (semi)landmark configurations were applied to all targets using Viewbox 4. The template repeatability (i.e., high intra‐observer agreement) and reproducibility were tested. Repeatability was assessed by having the same observer apply the template three times on different days. Reproducibility was tested by having two different expert observers apply the same template (C.F. and G.B.). Procrustes distance was used to calculate the distance between the landmark configuration. Based on this analysis, the intra‐ and interobserver errors were minimal (< 0.005), confirming the high repeatability and reproducibility of this landmark configuration. Semilandmarks were then allowed to slide recursively to minimize thin‐plate‐spline bending energy (Slice 2006) between the template and targets, and to make them geometrically homologous (Gunz and Mitteroecker 2013; Mitteroecker et al. 2013). Coordinates were then registered with a Generalized Procrustes Analysis (GPA) using the R (R Core Team 2021) package geomorph 3.3.1 (Adams and Otárola‐Castillo 2013). Size was then removed (centroid size, CS = 1), and specimens were translated and rotated. Finally, semilandmarks were allowed to slide against recursive updates of the Procrustes consensus (Rohlf and Slice 1990).

An exploratory shape space Principal Components Analysis (PCA) was performed on the Procrustes coordinates via the R package Morpho 2.8 (Schlager 2017). Shapiro Normality and Levene tests were carried out on the first three principal components (PCs) to evaluate the data distribution and its homoscedasticity, respectively. Parametric or non‐parametric tests (Analysis of Variance, ANOVA, or Kruskal–Wallis rank‐sum test, respectively) were performed as necessary to assess variance between group means along the first three PCs. Pearson's product–moment correlation was carried out to evaluate if shape variations were related to size (i.e., the natural logarithm of the CS). Procrustes form space was investigated to find any variations of size and shape through PCA by adding the natural logarithm of CS as an additional variable to Procrustes shape coordinates (Klingenberg 2016; Mitteroecker et al. 2004, 2013). This procedure reduces shape variation in a few dimensions while preserving size information (Mitteroecker et al. 2004).

#### 2.2.2 Trabecular Analyses

The segmented micro‐CT image data were then analyzed using Medtool 4.3 (Dr. Pahr Ingenieurs e.U, 2017). For each calcaneus, trabecular and cortical bone were separated following the protocol of Gross and colleagues (Gross et al. 2014). Opening and closing filters of varying kernel sizes (3–5 mm) were applied to the segmented data, followed by a filling procedure designed to algorithmically delineate the interior border of the cortical shell (Pahr and Zysset 2009). If the excessive cortical porosity of the bone made this procedure difficult, an iterative dilation‐and‐erosion cycle was applied to produce a closed cortical shell. Then, an outer mask (i.e., external surface) and inner mask (i.e., inner surface) were subtracted from the original segmentation to separate cortical bone from trabecular bone (Gross et al. 2014; Stephens et al. 2016, 2018). Finally, the computational geometry algorithms library CGAL (www.cgal.org) was used to generate a tetrahedral mesh of trabecular bone using the Delauney triangulation (Delaunay 1934; Gross et al. 2014; Komza and Skinner 2019).

A 5 mm spherical volume moving along a background grid of 2.5 mm spaced nodes was used to quantify bone volume fraction (BV/TV) and degree of anisotropy (DA) on the segmented volume (Gross et al. 2014; Pahr and Zysset 2009). The mean intercept length (MIL) approach (Komza and Skinner 2019; Odgaard 1997), which gave results for first, second, and third eigenvectors and eigenvalues, was used to calculate DA. Then, the fabric DA was calculated (1 − [eigenvalue 3/eigenvalue 1]) and scaled between 1 and 0 (1 indicates a highly anisotropic pattern and 0 an isotropic one). Results were interpolated to the centroid of the elements within the tetrahedral meshes, and colormaps were used to visualize patterns in Paraview 3.14.1 (Sandia Corporation, Kitware Inc). Mean trabecular thickness (Tb.Th, mm), mean trabecular number (Tb.N), and mean trabecular separation (Tb.Sp, mm) were also quantified (Hildebrand and Rüegsegger 1997; Stephens et al. 2018). A piecewise regression via the R package “segmented” (Muggeo 2008) was run to investigate significant changes in the slope of trabecular structure in relation to age, following Saers et al. (2022a, 2022b).

## 3 Results

### 3.1 External Morphology

**FIGURE 2:**
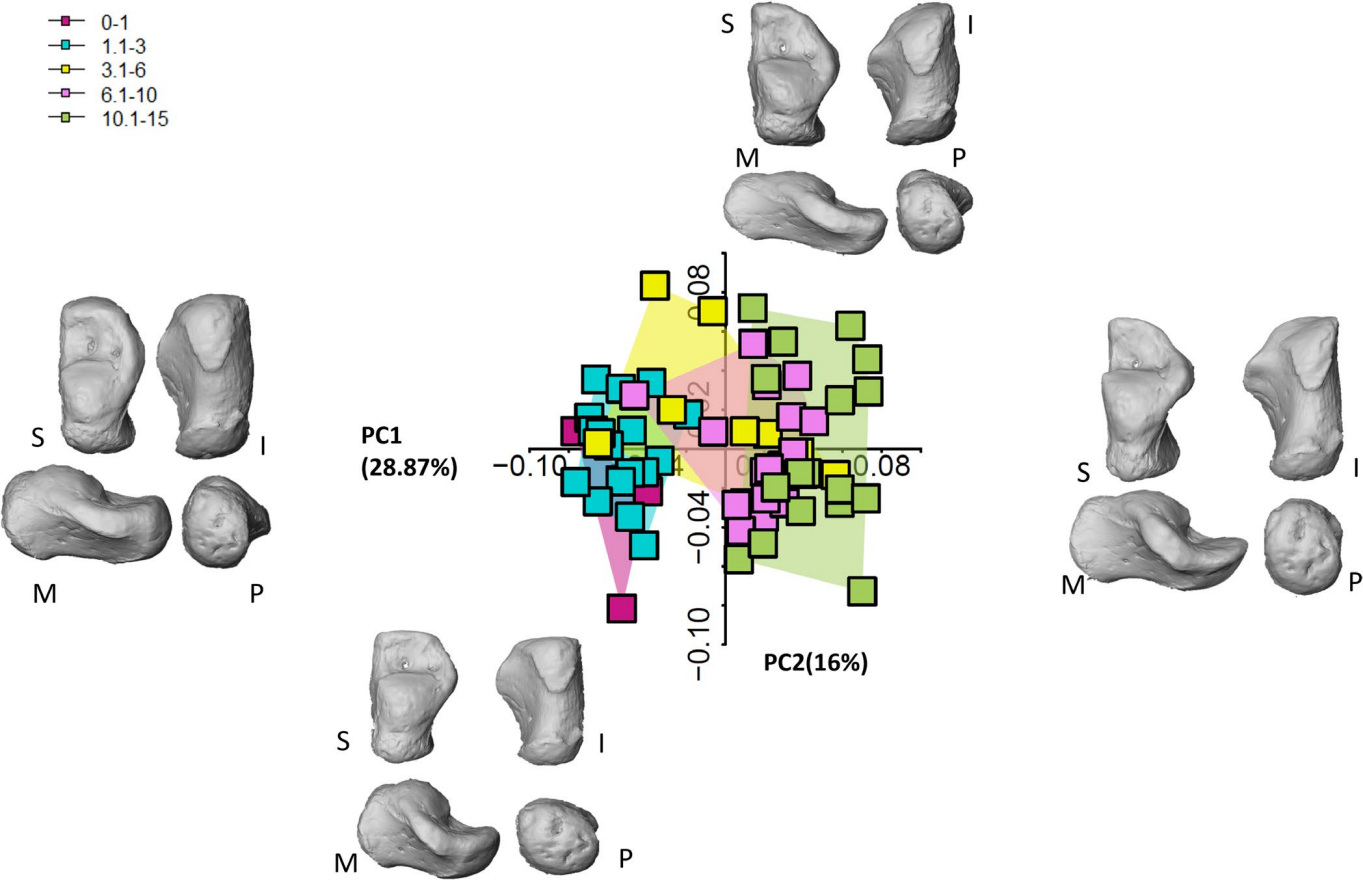
2D plot in shape space and morphological shape deformations of the first two PCs. It is possible to appreciate a separation in the 3.1‐6‐year group (yellow squares) and a separation along PC2 in the oldest age group (green squares). Calcanei are shown in superior (S), inferior (I), medial (M), and proximal (P) views.

The first three PCs explain 55% of the total variance in shape space (Figure 2, but see also Figures S1, S2, S3). Table 5 summarizes the morphological variations in shape space.

**TABLE 5:** Morphological variation in shape space.

	Negative scores	Positive scores
	Youngest age groups (1.1–3 and 3.1–6 years) minus the individuals from Bologna in the 3.1–6‐year group	The oldest individuals (6.1–10‐ and 10.1–15‐year groups) plus the individuals from Bologna belonging to the 3.1–6‐year group)
PC1 (28.38%)	Immature morphology, with an overall compact aspect. The anterior and medial talar facets are not developed yet, while the posterior is distinguishable but still very immature in shape. The cuboid facet is rounded and convex, while the sustentaculum tali is present but very small and immature (i.e., medial shelf) The lateral and medial processes are slightly visible.	More mature morphology, slender and more robust, with a prominent posterior calcaneal tuber (metaphyseal). Anterior, medial, and posterior talar facets are forming, with the posterior more defined and mature than the other two. The sustentaculum tali has changed its shape and size, assuming a more adult‐like morphology. The cuboid facet has assumed a more adult‐like shape, without the roundness of the immature one. The lateral and medial processes are well‐defined.
PC2 (15.9%)	The calcaneus is short in respect to length, and the cuboid facet is round and convex.	The calcaneus is narrow and elongated, but still robust. The cuboid facet lost its convexity.
PC3 (10.6%)	The sustentaculum tali is short and not developed yet.	The sustentaculum tali increased in size and displayed a more mature morphology.

Only PC1 departs from a normal distribution (Shapiro–Wilk normality test: *W* = 0.92, *p‐* value = 0.001), while Levene tests attest to the homogeneity of variance of PC2 and PC3 (PC1: *F*‐value = 2.6, *p*‐value = 0.04; PC2: *F*‐value = 1.08, *p*‐value = 0.3; PC3: *F*‐value = 1.7, *p*‐value = 0.15). PC1 alone explains 28.4% of the total variance, while PC2 and PC3 account for 16% and 9% of the total variance, respectively. PC1 is highly correlated with size (*r* = 0.86, *p*‐value < 0.001), capturing the allometric differences in morphology. Only PC1 and PC3 scores highlight significant differences between age classes (PC1: Kruskal–Wallis—chi‐squared = 45.18, df = 4, *p*‐value = < 0.001; PC2: ANOVA—df = 4, *F*‐test = 1.2, *p*‐value = 0.31; PC3: ANOVA—df = 4, *F*‐test 2.8, *p*‐value = 0.02). A Dunn's test on PC1 scores indicates significant differences between the two youngest classes and the oldest class, which was expected, and between the 1.1–3‐year and 6.1–10‐year classes (Table 6). There are no significant differences between consecutive age classes except for one pair (1.1–3 and 3.1–6). A Tukey's test on PC2 scores did not identify any significant differences between consecutive age classes (Table 7). In morphospace (Figure 2), it is possible to appreciate a separation between the two youngest groups (0–1 and 1.1–3) and the two oldest groups (6.1–10 and 10.1–15). As for the middle age group (3.1–6), there is a clear separation within the group, whereby individuals from Velia and Parma (known age‐at‐death) plot with the two youngest groups and individuals from Bologna plot with the two oldest groups. This separation within the group is not linked directly to the individuals' age, as in this group the individuals' age‐at‐death are predominantly distributed around the age of five within the broader age range category, with the sole exception of two individuals from Bologna (3 and 6, with known age‐at‐death) and an individual from Velia (4 years). Another instance of intragroup separation occurs in the oldest age group along PC2.

**TABLE 6:** Dunn's test PC1. *p* values.

Age groups	0–1	1.1–3	3.1–6	6.1–10
1.1–3	1.00	—	—	—
3.1–6	0.6	0.10	—	—
6.1–10	0.07	**0.0003**	1.00	—
10.1–15	**0.0009**	**> 0.001**	0.11	0.49

*Note:* *p*‐Value adjustment method: Bonferroni. Statistically significant *p*‐values are in bold.

**TABLE 7:** Tukey's test PC3—pairwise comparison between age groups.

	Difference	Lower	Upper	*p*‐Value adjusted
1.1–3 vs. 0–1	−0.03	−0.07	0.01	0.25
3.1–6 vs. 1.1–3	−0.03	−0.7	0.1	0.30
**6.1–10 vs. 0–1**	−0.04	−0.08	−0.005	**0.01**
10.1 vs. 15–0‐1	−0.03	−0.07	0.003	0.09
3.1–6 vs. 1.1–3	−0.0008	−0.03	0.029	0.99
6.1–10 vs. 1.1–3	−0.01	−0.04	0.008	0.34
10.1–15 vs. 1.1–3	−0.006	−0.03	0.01	0.93
6.1–10 vs. 3.1–6	0.016	−0.04	0.01	0.57
10.1–15 vs. 3.1–6	0.006	−0.03	0.02	0.97
10.1–15 vs. 6.1–10	0.009	−0.01	0.03	0.79

*Note:* Statistically significant *p*‐values are in bold.

PC1 negative scores describe a very immature morphology, corresponding to the youngest individuals. The overall aspect of the immature calcaneus is its more compact shape, not being as narrow and slender as the mature one, which elongates proximodistally. In contrast, positive scores describe a mature morphology, characterized by a completely formed sustentaculum tali and the presence of a sustentacular groove. The lateral and medial plantar processes are visible and completely formed in the more mature morphology. The sustentaculum tali is small and subtly defined in the youngest calcanei, which is even more appreciable from the inferior view. The inferior surface is less curved in the youngest individuals, while the oldest individuals display a more curved surface when seen in medial or lateral views. This is attributable to the growth in height of the whole calcaneal body and, most importantly, to the calcaneal tubercle which becomes more robust with age. While the tubercle is discernible in the youngest cohort, it is narrower and less prominent in medial/lateral views. In lateral view, it is possible to appreciate the growth of the posterior talar surface, which changes slightly in orientation and becomes more vertically oriented and slightly more convex, while in posterior view it is possible to appreciate an enlargement in a lateral‐medial direction. The anterior aspect of the calcaneus is more squared in superior view, while it changes to a blunter medial angle and a more pointed lateral extremity with age. The cuboid facet displays numerous changes in shape with age. In the youngest cohort, this joint surface is already well‐defined and is convex and rounded, while it becomes increasingly concave and deeper in older groups. The subtalar articular facets are also clearly discernible in the youngest cohort, showing well‐defined margins. The medial and lateral plantar processes are also distinguishable even in the youngest individuals. The peroneal trochlea, on the other hand, appears only in the oldest cohort.

PC2 and PC3 scores also describe a morphology that transforms from a more compact bone to a more narrow and elongated morphology with age, as it assumes adult morphology.

**FIGURE 3:**
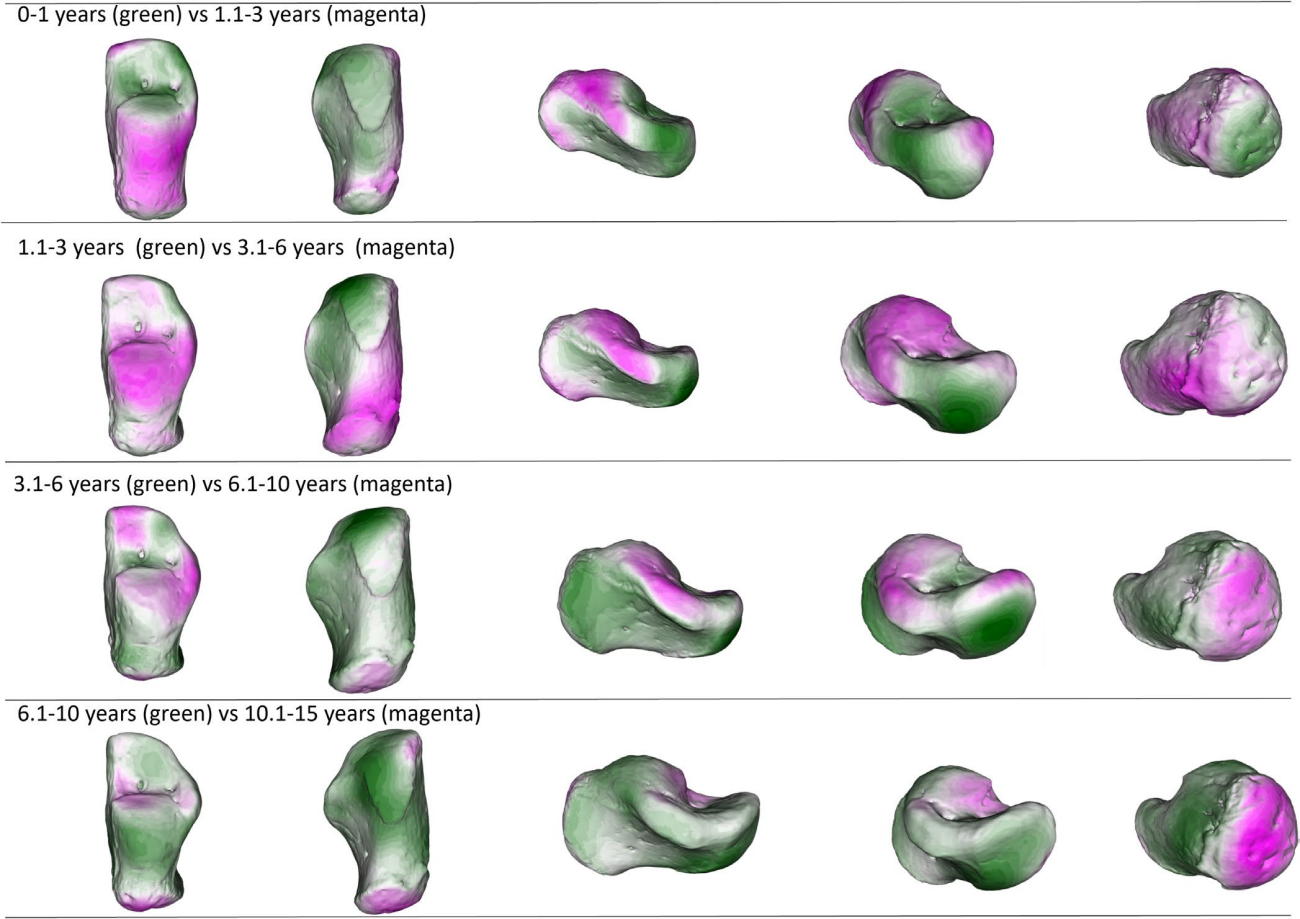
Displacement heatmaps showing calcaneal shape differences between age groups means. Green represents the younger age group in each comparison, while magenta represents the older age group in each comparison. Colors mapped to the older age group in each comparison depict vertices that extend beyond (magenta) the mesh (which represents the younger age group in each comparison). The distance is calculated by superimposing the meshes of the two age groups for each comparison. For instance, the magenta color of the calcaneal body in the first comparison (0–1 year vs. 1.1–3 years) means that the 1.1–3 years group extends beyond the calcaneal body of the 0–1 year group. After the onset of independent walking, there is a proximodistal elongation of the calcaneal body and a slight increase in the medial projection of the sustentaculum tali. The cuboid facet angle starts to change in its lateral part. With the achievement of more mature locomotor kinematics, between 3 and 6 years of age, the posterior talar facet changes in orientation and lateral‐medial elongation. The calcaneal body and the sustentaculum tali continue to lengthen, which increases in size and morphology. After six years of age, mostly all remaining changes are concentrated on the metaphyseal area, that is, calcaneal tuberosity, and lateral part of the cuboid facet. The posterior talar facet continues to develop, and the anterior talar facet undergoes major developmental changes as the sustentaculum tali develops.

When group average are considered (Figure 3, but see also Figures S4, S5, S6, and S7), it is possible to identify some developmental clusters. The 1.1–3‐year group displays an immature calcaneus, with very few differences from the 3.1–6‐year group, that is, the slight medial expansion of the sustentaculum tali. The lack of significant differences between these two groups underscores their similarity. On the other hand, differences are more apparent between the 3.1–6‐ and 6.1–10‐year groups. The sustentaculum tali has started changing its morphology, as well as its dimension across the groups. Also, the anterior part of the calcaneus, that is, the future anterior talar facet, is changing its morphology, with a more defined and adult‐like shape. The calcaneus appears to be globally more robust. The cuboid facet has started to change, but it is only in the next age group, 10.1–15, that its shape resembles the adult shape, with its characteristic hourglass‐like morphology, as described by Bojsen‐Møller (1979). The anterior talar facet is now well‐defined, and the posterior talar facet has shown its peculiar flat morphology.

In form space, the first three PCs account for 97.1% of the total variance. PC1 alone accounts for 95.3% of the total variance, which is explained by a high correlation with size (*r* = 0.98, *p*‐value = > 0.001), as expected (Figure S2). PC2 and PC3 account for 0.1% and 0.6%, respectively. In morphospace, individuals are flattened on the PC1 axis, showing an ontogenetic trajectory (“growth‐axis”).

### 3.2 Internal Morphology

**FIGURE 4:**
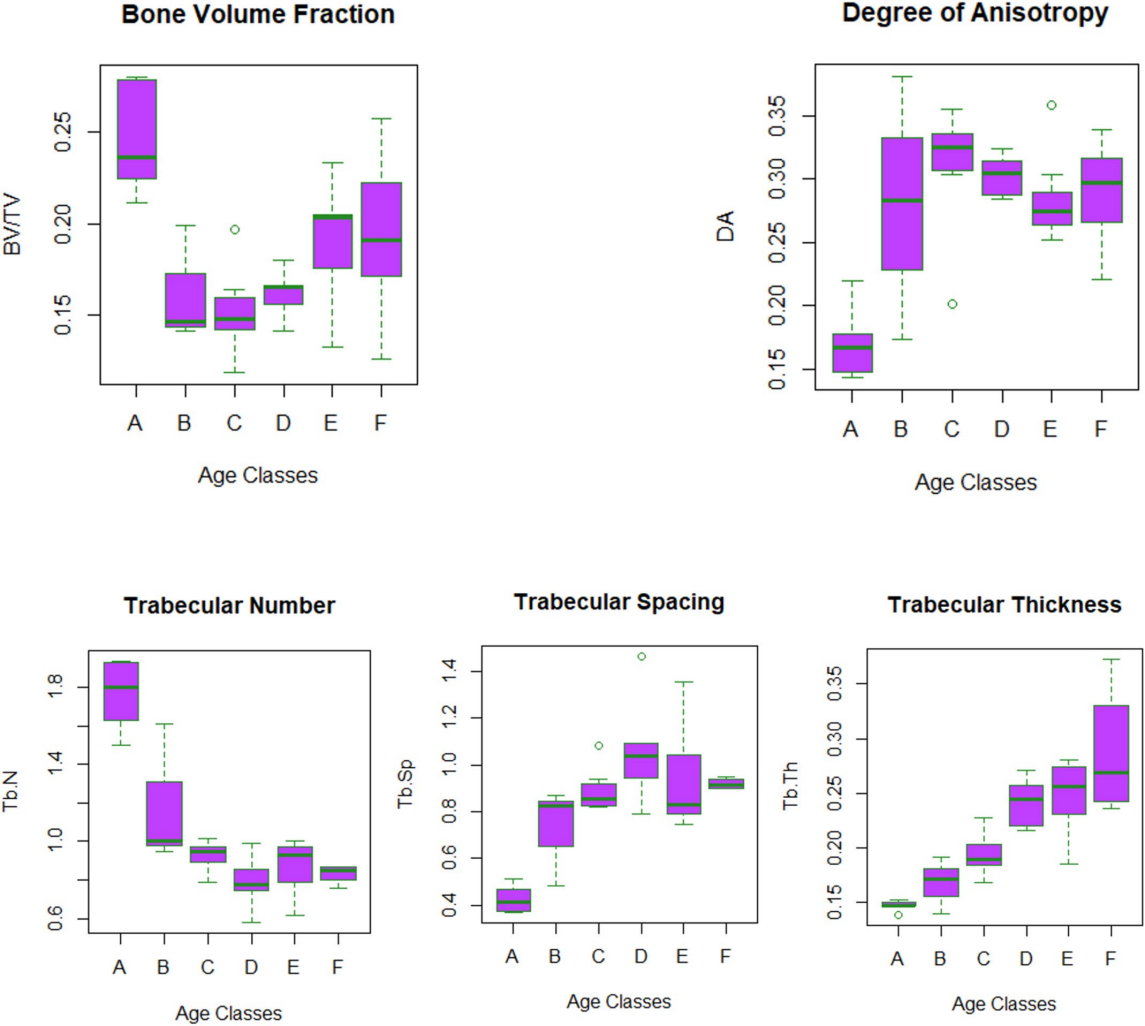
A = perinatal; B = 0–1 year; C = 1.1–3 years; D = 3.1–6 years; E = 6.1–10 years; F = 10.1–15 years. Boxplots of trabecular bone structural properties per age group. The bar indicates the median, the box shows the interquartile range, while whiskers represent minimum and maximum values. Outliers are indicated by dots.

**FIGURE 5:**
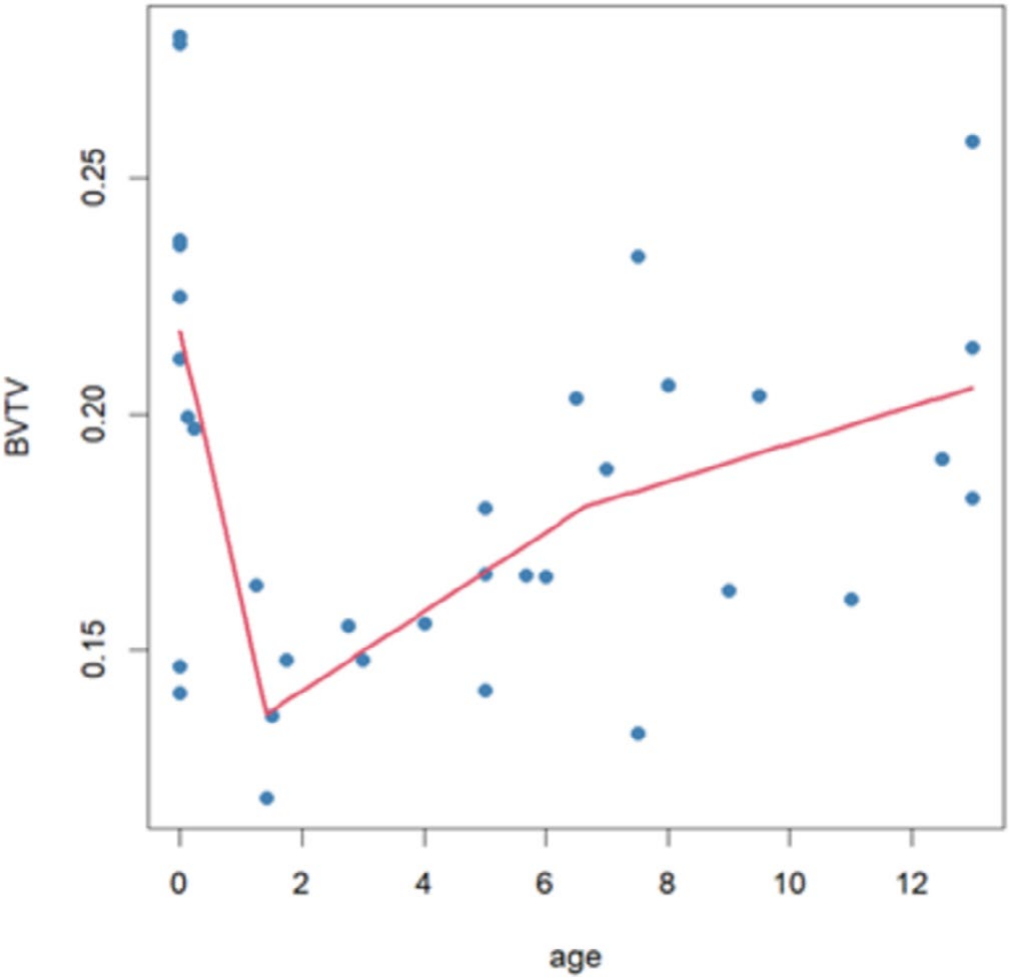
BV/TV trajectory. It is possible to identify three main developmental phases, in line with Saers et al. (2022a, 2022b). The individuals have shown here come from the Osteological Collection of the University of Bologna, with known age‐at‐death.

**FIGURE 6:**
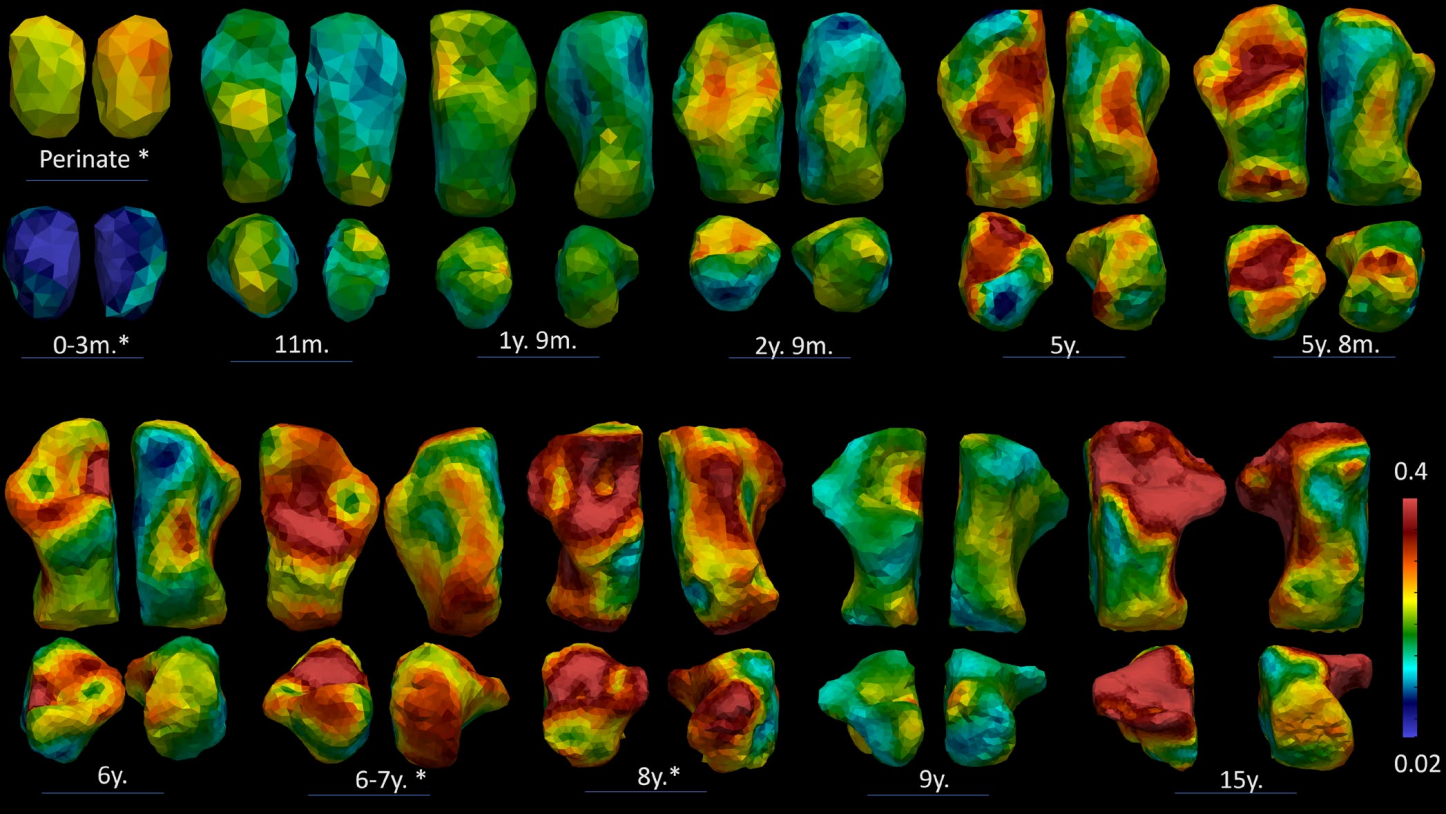
Bone Volume Fractions. The representative individuals, that is, closest to the mean of group cluster, are represented in four views, clockwise direction: Superior view, plantar view, distal view, and proximal view. Perinate and the 0–3‐month‐old individual are only represented in superior and plantar views. The individuals here represented are from Bologna; individuals with * are from Velia.

**FIGURE 7:**
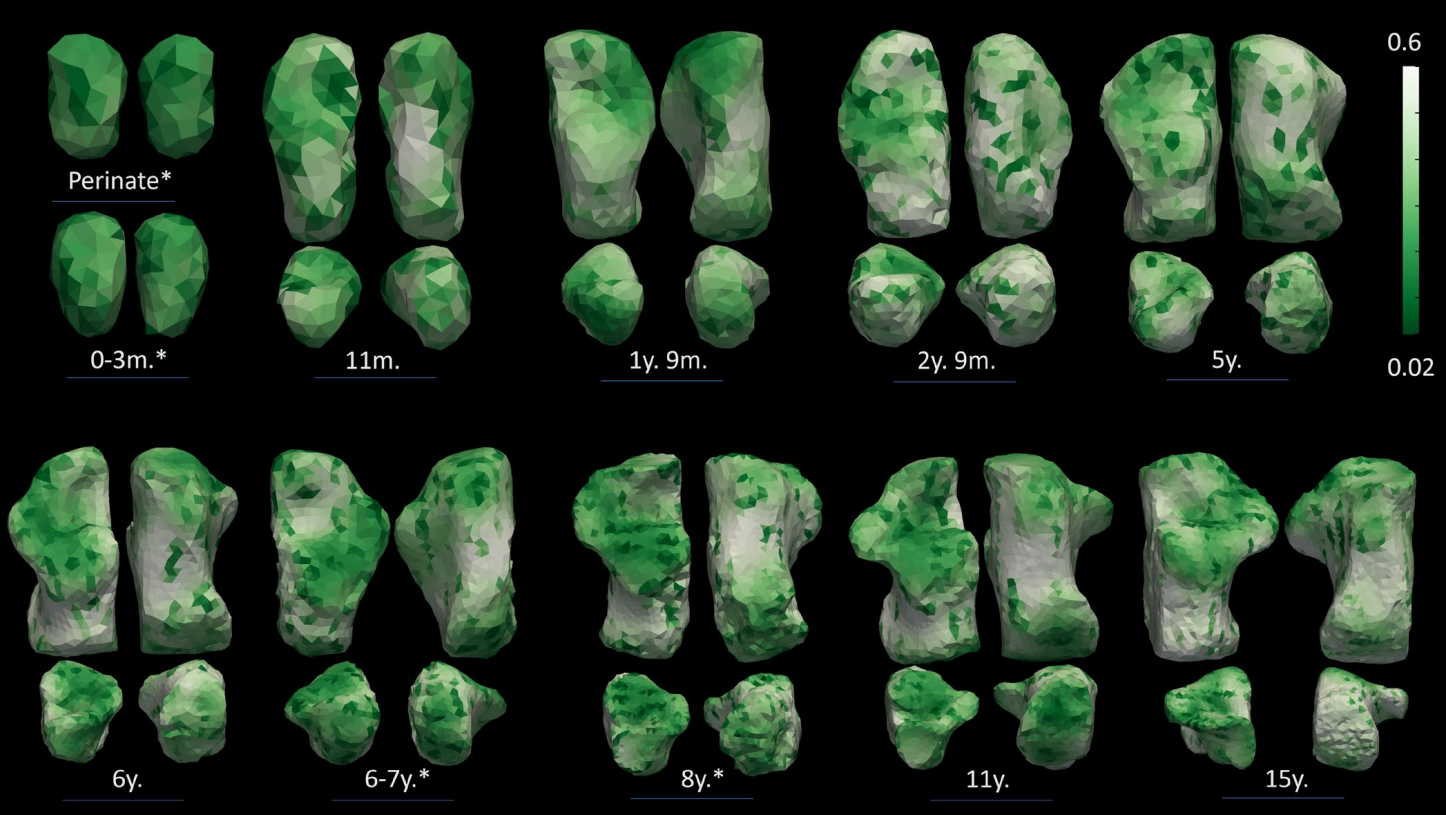
Degree of Anisotropy. The representative individuals, that is, closest to the mean group cluster, are represented in four views, clockwise direction: Superior view, plantar view, distal view, and proximal view. Perinate and the 0–3‐month‐old individual are only represented in superior and plantar views. The individuals here represented are from Bologna; individuals with * are from Velia.

Mean trabecular values for each age class and trabecular trends are shown in Table 8, and Figures 4, 5, 6, and 7. In the perinates cohort, the number of trabeculae is the highest of the sample, accompanied by the lowest values for separation and thickness. These values describe a very dense and packed structure, with numerous and thin trabeculae very closely assembled in the group. This pattern changes as age increases, showing a drop in Tb.N from birth until 6 years of age. After age 6, there is a slight increase in Tb.N during adolescence (up to 15 years). The decrease in Tb.N is accompanied by a corresponding increase in Tb.Sp and a slight increase in Tb.Th as the space between trabeculae increases, their number reduces, and trabeculae thicken. This trend is constant until 6 years of age when there is a slight decrease in Tb.Sp as Tb.Th continues to rise.

**TABLE 8:** Group averages, medians, and standard deviations of Tb.N, Tb.Sp., and Tb.Th, BV/TV and DA.

Age class	Tb.N mean (median, SD)	Tb.Sp mean (median, SD)	Tb.Th mean (median, SD)
Perinates	1.76 (1.79, 0.17)	0.42 (0.41, 0.004)	0.14 (0.14, 0.004)
0–1	1.18 (1.0, 0.36)	0.72 (0.82, 0.26)	0.16 (0.17, 0.02)
1.1–3	0.92 (0.94, 0.07)	0.89 (0.85, 0.01)	0.19 (0.189, 0.18)
3.1–6	0.78 (0.77, 0.13)	1.06 (1.03, 0.02)	0.24 (0.24, 0.21)
6.1–10	0.86 (0.93, 0.15)	0.94 (0.83, 0.03)	0.24 (0.25, 0.35)
10.1–15	0.83 (0.85, 0.05)	0.91 (0.91, 0.06)	0.28 (0.269, 0.06)

Age class	BV/TV mean (median, SD)	DA mean (median, SD)	
Perinates	0.24 (0.23, 0.02)	0.17 (0.16, 0.02)	
0–1	0.16 (0.14, 0.03)	0.27 (0.28, 0.10)	
1.1–3	0.15 (0.14, 0.02)	0.30 (0.32, 0.05)	
3.1–6	0.16 (0.16, 0.01)	0.30 (0.30, 0.01)	
6.1–10	0.19 (0.20, 0.03)	0.28 (0.27, 0.03)	
10.1–15	0.19 (0.19, 0.04)	0.28 (0.29, 0.04)	

As for BV/TV and DA, values show a similar pattern to Tb.N, Tb.Sp, and Tb.Th. BV/TV exhibits the highest values in the perinates group. Then, values drop after birth and start slightly increase again after 1 year of age. After 3 years, the increase is steadier, but bone volume fraction values do not reach prenatal values. DA, on the contrary, shows the lowest values in the perinates cohort, describing a rather isotropic structure. DA values rise until 6 years of age. After 6 years, DA slightly decreases and remains stable until 15 years of age.

The highest BV/TV values in the perinates are seen beneath the inferior‐medial surface. In the 0–1‐year‐old group, this pattern changes as the highest values occur beneath the posterior and medial surfaces, anterior talar facet, and posterior talar facet. Similarly, in the 1.1–3‐year‐old group, the highest values are seen beneath the sulcus calcanei and subtalar joints, and the posterior‐medial surface. In the 3.1–6‐year‐old group, the highest values are recorded beneath the sulcus calcanei, while in the 6.1–10‐year‐old group, the highest values occur beneath the subtalar joints and posterior medial surface. In the oldest group, 10.1–15, while values are slightly lower, the highest values are seen beneath the posterior talar facet, calcaneocuboid facet, and the posterior metaphyseal surface. In only one individual the calcaneal epiphysis was fused, displaying higher BV/TV values in the calcaneal tuberosity.

As for DA, the highest values are seen beneath the posterior talar facet and the calcaneocuboid joint. After 3 years, DA values increase also beneath the inferior calcaneal surface.

Finally, the plot shown in Figure 6 highlights two slopes in BV/TV trajectory, identifying three main developmental phases.

## 4 Discussion

Overall, our results are in line with previous investigations (calcaneus: Saers, Ryan, and Stock 2020, Saers et al. 2022a, 2022b; talus: Figus et al. 2022; Figus, Sorrentino, et al. 2023; Figus, Stephens, et al. 2023). The most functionally relevant morphological adaptations are observed before the age of six when the biggest locomotor milestones take place (Table 9). As seen in the talus, the external calcaneal morphology exhibits a slower timeline of development, and is less informative, due to the genetic blueprint, than the internal structure. The external shape analysis shows great allometric‐related changes along PC1, especially between 1.1–3‐ and 6.1–10‐year groups. On the whole, our results suggest that trabecular architecture in the calcaneus responds to mechanical loading during gait development, as also suggested by previous studies on biomechanically stressed anatomical areas (Boyle et al. 2018; Figus et al. 2022; Figus, Sorrentino, et al. 2023; Figus, Stephens, et al. 2023; Ryan and Krovitz 2006; Saers, Ryan, and Stock 2020; Saers et al. 2022b). When less strained areas are taken into account, for example, the humerus, the BV/TV pattern is different, for example, Chevalier and colleagues (2021) found that BV/TV values decrease after birth, but the values remained relatively constant until 14 years of age.

**TABLE 9:** Description of the internal and external variations.

Youngest age groups (1.1–3‐ and 3.1–6‐year groups) minus the individuals from Bologna in the 3.1–6‐year group		The oldest individuals (6.1–10‐ and 10.1–15‐year groups) plus the individuals from Bologna belonging to the 3.1–6‐year group)	
External morphology	Internal morphology	External morphology	Internal morphology
Immature morphology, with an overall compact aspect. The anterior and medial talar facets are not developed yet, while the posterior is distinguishable but still very immature in shape. The cuboid facet is rounded and convex, while the sustentaculum tali is present but very small and immature (i.e., medial shelf) The lateral and medial processes are slightly visible.	BV/TV: highest magnitude on the medial side and medial shelf, then shifts also to the posterior talar facet. DA: highest magnitude in the posterior talar facet and calcaneocuboid join. Then, after 3 years, DA increases in the inferior surface.	More mature morphology, slender and more robust, with a prominent posterior calcaneal tuber (metaphyseal). Anterior, medial, and posterior talar facets are forming, with the posterior more defined and mature than the other two. The sustentaculum tali has changed its shape and size, assuming a more adult‐like morphology. The cuboid facet has assumed a more adult‐like shape, without the roundness of the immature one. The lateral and medial processes are well‐defined.	BVTV: highest magnitude in the floor of the sinus tarsi. DA: highest magnitude in the posterior talar facet and the calcaneocuboid joint.

### 4.1 External Morphology

*Perinates and 0–1‐year group*: Negative scores describe an overall immature morphology, corresponding to the youngest individuals, that is, 0–1‐year, with a compact morphology that has not yet developed the typical narrow and slender aspect of the adult human calcaneus. This is consistent with a bone that is not routinely subject to bearing body weight loads.

*1.1–3‐year group*: During this period, at around 12 months, the foot starts to be loaded more as toddlers engage in independent walking. Around this time during development, body weight increases to twice (or slightly more) the initial birth weight (Wells and Cole 2002). The 1.1–3‐year group still displays an immature calcaneus that resembles the calcaneal shapes of the 1.1–3‐year group and the non‐Bolognese individuals included in the 3.1–6‐year group, for example, the individuals from Velia and Parma (with known age‐at‐death). During this period of development, there are slight changes in the medial expansion of the sustentaculum tali. The calcaneal tubercle becomes recognizable, but it is not as thick as in the adult condition. The posterior talar facet is less vertically oriented in this age phase, while the anterior part of the calcaneus appears more squared in superior view, describing a still immature distal configuration of the anterior talar facet and cuboid facet. Specifically, the former has no defined margins, while the latter exhibits a convex and rounded shape, with no or little asymmetry. Early development of the anterior calcaneal facet in the talus (Figus et al. 2022) is not reflected in the calcaneus, as development in the anterior talar facet is delayed compared to development in the posterior talar facet.

*3.1–6‐year group*: The most striking morphological differences are displayed between the 3.1–6‐ and 6.1–10‐year groups. The calcaneus has elongated in its proximodistal axis, developing a narrow and slender shape, and its peculiar robustness starts to be recognizable.

The distinct division in morphospace between individuals comprising the 3.1–6‐year group does not seem to be related solely to age, as individuals' ages are rather narrowly distributed within the group. Instead, the separation appears to be mainly driven by population differences, with a distinct separation between Bologna (individuals plot with the oldest individuals in the sample) and Velia and Parma (individuals plot with the youngest). Morphology characterizing these two blocks is quite distinct, with the latter displaying a more immature morphology, that is, a shorter, larger calcaneal body, and less developed posterior talar and cuboid facets. The more mature morphology displayed by the Bologna group, with a narrower and more slender calcaneal body coupled with slightly more developed features, may be explained by differences in social background and, probably, genetics and footwear. Nutrition habits may have also differed between the modern and urban Bologna cohort versus the Velia group, which is an Imperial Roman cohort. Differences between Parma and Bologna, though, may be comparatively less straightforward, since only one individual from Parma is present in this cohort. It is important to emphasize that, while the age‐at‐death of individuals in the Velia sample is estimated, the exact age‐at‐death is available for individuals from Parma and Bologna. This difference may indicate a major shift in the calcaneal morphology around 5 years of age. This would be consistent with the development of the longitudinal arch of the foot and more adult‐like foot kinematics during bipedalism, or to differences in growth due to environment, diet, and genetics. According to Holowka et al. (2022), differences in the terrain, that is, more urban versus more impervious ground, might have influenced the way children walked. A more uneven substrate might have required more balance control, that is, a prolonged flatter foot contact to increase the plantar surface area and have greater sensory feedback, ultimately influencing the morphological development. Hypothesis 1a has been partially fulfilled, as it is possible to appreciate a separation between a more immature and more mature morphology around 3 years.

*6.1–10‐year and 10.1–15‐year groups*: Positive PC1 scores account for a more mature and almost adult morphology, which corresponds to the oldest individuals in the overall sample (the Bologna individuals in the 3.1‐6‐year group, and individuals in the 6.1–10‐ and 10.1–15‐year groups). Thus, until age 8, the feet of children have a proportionately wider breadth than those of older children. After about 8 years, foot proportions begin to become more like those of adults. This might reflect developmental maturation in locomotor kinematics, as the change in foot proportion may create a more effective lever for generating force at push‐off. During this developmental period, the external morphology of the calcaneus also shows changes consistent with a foot that sustains compressive loads associated with heel strike.

The sustentaculum tali started to form after 6 years and is completely formed, as well as the sustentaculum groove, by 10.1–15 years. The anterior talar facet is developing, displaying a more defined and adult‐like shape morphology. On the other hand, the lateral and medial plantar processes are visible and completely formed in all the individuals exhibiting positive scores. The calcaneal tubercle becomes more robust. The posterior talar facet becomes more vertically oriented, slightly more convex, and expands in a lateral‐medial direction. The distal aspect of the calcaneus shows a blunter medial angle and a more pointed lateral extremity.

The cuboid facet becomes increasingly more asymmetric with age, developing its peculiar aspect defined by Bojsen‐Møller (1979) as resembling an hourglass. Asymmetry and convexity of this facet are thought to ease the closed‐packed position of the calcaneocuboid joint during inversion, contributing to an increased stiffness during toe‐off (Bojsen‐Møller 1979; Harper, Ruff, and Sylvester 2021), and is subjected to compressive loads (Giddings et al. 2000). This is suggestive of a foot already accustomed to adult‐like locomotor kinematics. The depth of the cuboid facet, which assumes a more mature aspect around 6 years, allows for increased midfoot flexibility by facilitating inversion/eversion (Holowka et al. 2017a). This suggests the development of a midfoot with some degree of stiffness, as the adult shape displays a relatively convex cuboid facet morphology and a relatively flat posterior talar facet, which are already visible in the oldest sample (10.1–15 years). Hypothesis 1b has been fulfilled, as it is possible to record the presence of a more mature morphology on the cuboid facet during this phase.

Another interesting separation is visible within the 10.1–15‐year group, notably along PC2. Individuals from Bologna (except for one), Beli Manastir, and Cagliari (except for one) plot towards negative PC2 values, while all individuals from Sassari, Campochiaro Morrione, Parma, and St. John plot towards positive PC2 values. Interestingly, when the sex variable is considered (in the control sample, with known sex), all female individuals plot as positive values, with the sole exception of two male individuals. The morphological difference is linked to the robustness of the calcaneal body, with shorter and larger calcanei (negative values) separated from more slender calcanei (positive values). This difference may be explained by the fact that this age class includes adolescent individuals. Adolescence is a particular period, where very rapid growth is accompanied by sexual maturation. The fact that all the female individuals plot towards more positive values, that is, exhibit more mature morphology, may be the consequence of accelerated female growth compared to contemporary males. The growth spurt may be influenced by many factors, such as sex, correct food intake, cultural background, and environment. Nonetheless, dimorphic differences have been found in the adult calcaneus too, particularly in dimensions and expansion of the medial and lateral plantar processes.

Differences between populations with dissimilar behaviors and backgrounds have been noted by previous studies: calcaneal anteroposterior length has been shown to differ between Medieval and Post‐Medieval populations in relation to footwear (Albee 2022), while a relatively wider anterior/middle talar facet in mobile populations was linked to relatively higher loading through the calcaneus (Harper, Ruff, and Sylvester 2022). More recently, Harper (2023) found that modern populations (mid‐20th century industrialized populations), supposedly characterized by more sedentary behavior and/or different footwear (more rigid), exhibited a more gracile (relatively mediolaterally narrower and anteroposteriorly longer) calcaneal tuber compared to earlier industrialized populations. The relatively mediolaterally wide and anteroposteriorly short calcaneal tuber is thought to be an adaptation for the transmission of forces through the posterior calcaneus during heel strike (Prang 2015). In addition, the tall posterior calcaneal surface has been linked to bipedalism, as it is suggestive of an adaptation to a potential increase in magnitude and bending stresses (Harper, Ruff, and Sylvester 2022; Su et al. 1999).

More mobile populations have a mediolaterally wider posterior talar facet. During growth, the mediolateral enlargement of this facet may be linked to a reduction in peak stress and strain due to the wider surface area of the facet, which may more effectively spread the load distribution across the subtalar joint (Harper 2023; Ruff 1988). A larger calcaneus may also be linked to an increase in body size (Harper 2023). These possibilities highlight the potential plastic relationship between foot morphology and environment/behavior (Albee 2022; DeMars et al. 2021; Harper 2023; Sorrentino, Stephens, et al. 2020).

### 4.2 Internal Morphology

Internal morphology shows high variability in the sample. As hypothesized, the number of trabeculae is highest in the perinatal cohort, together with the lowest values in trabecular separation and thickness.

*Perinates and 0–1‐year group*: As hypothesized in H2a, the perinatal and neonatal architecture is very dense, with many thin and closely packed trabeculae, and responds to a more demanding need for calcium (Acquaah et al. 2015). This not‐yet specialized morphology reflects the infrequency of the loads: at this age (< 1 year), the foot is mostly underloaded, as the infant starts is completely dependent on the caregiver (< 6 months) or starts engaging in crawling, standing on two legs, and walking with assistance (> 6 months), practices that usually intermingle for a period. As children grow and their locomotor kinematics mature, these values change. The highest BV/TV values in the perinates are seen beneath the inferior‐medial surface. In the 0–1 year group, this pattern changes as the highest values are beneath the posterior and medial surfaces, the anterior talar facet, and the posterior talar facet. During the first half of the first year of life, there is a rapid decrease in BV/TV values. The physiological postnatal bone loss is a well‐documented phenomenon, which is thought to be the product of different inputs, for example, the removal of unstrained or underloaded struts (Frost 2003; Pivonka, Park, and Forwood 2018), and a more demanding need for calcium during the postnatal phase (Acquaah et al. 2015). This leads to lower BV/TV and Tb.N values in the postnatal period, as displayed in our sample, and an accompanying increase in Tb.Th. A dense and rather isotropic pattern in the calcaneus may be, as suggested by Saers, Ryan, and Stock (2020), the product of endochondral ossification, with the struts laid down radially, that is, isotropically, from the center of ossification. Interestingly, Ryan and Krovitz (2006) and Gosman and Ketcham (2009) observed the opposite pattern in the femur and tibia, respectively, with bone initially laid down in a single direction, that is, anisotropically, except for in the proximal femur. These differences are likely related to differences in ossification. Long bones need to increase in length and trabecular bone is initially laid down in a more regular and anisotropic pattern. On the other hand, the calcaneus ossifies radially from a center of ossification in a more isotropic pattern, that is, more similar to the epiphyseal portion of the proximal femur.

*1.1–3‐year group*: Similarly, in the 1.1–3 year group, the highest BV/TV values are seen beneath the sulcus calcanei and subtalar joints, and the posterior‐medial surface.

Increased loading linked to the onset of unassisted walking, around 12 months, is probably the main factor responsible for the slow reversal in the BV/TV trend, for example, BV/TV stops declining and slowly increases again, even though the high perinatal values will not be attained. During this period, when the foot is loaded with more consistent walking, compressive bands start forming at the posterior talar facet and the calcaneocuboid joint (Saers, Ryan, and Stock 2020), which is supported by higher values of BV/TV visible in this area on the colormaps of 1.1–3‐ and 3.1–6‐year groups. Saers, Ryan, and Stock (2020) found that the posterior talar facet VOI, which experiences the highest mechanical loading during gait, also exhibited the highest BV/TV values. Our results are in line with this observation, as the same pattern has been observed in our sample. This suggests remodeling of trabecular struts as a response to high strain. H2b is not fulfilled, as we expected a greater increase in BV/TV values.

*3.1–6‐year group*: Trabecular number decreases while thickness and separation increase, until approximately 6 years of age. DA continues to increase until around 6 years of age when it finally reaches a plateau. This is accompanied by high BV/TV values in the proximal part of the calcaneus in all the groups except for 10.1–15‐ and 3.1–6‐year groups. This can be linked to the development of a proper heel strike, even though some amount of force may be absorbed by the cartilage (Saers, Ryan, and Stock 2020) and heel fat pad. This trend of increasing BV/TV continues, and trabecular bone appears to be more influenced by increases in body weight as locomotor kinematics mature around and after 6 years of age, with the posterior talar facet displaying the highest values in all the age groups. The regions with the lowest DA values are the posterior talar facet and calcaneocuboid, while higher values are displayed by the inferior and proximal parts of the calcaneus. Saers, Ryan, and Stock (2020) noticed that the VOI located near the plantar ligament region showed a delayed increase in DA compared to the other two VOIs of their study (i.e., the posterior talar facet and calcaneocuboid joint). They attributed this to two main events: (1) the plantar ligament region ossifying later than the other two regions, and (2) the longitudinal arch developing around 6 years of age. The development of the longitudinal arch may contribute to increased tensile stress on the plantar ligaments. Anisotropy steadily increases after 5 years of age, possibly reflecting less variable and more stereotypical bipedal gait kinematics (Raichlen et al. 2015; Zeininger, Shapiro, and Raichlen 2017; Zeininger et al. 2018). Hypothesis 2c has been fulfilled, as DA and BV/TV increase, in response to a more stereotypical pattern of locomotion.

*6.1–10‐year and 10.1–15‐year groups*: these age periods are characterized by a slight decrease in Tb.N coupled with increases in Tb.Sp and Tb.Th. Both BV/TV and DA show a similar pattern. In the 6.1–10‐year group, the highest values are beneath the subtalar joints and posterior medial surface. In the oldest group, 10.1–15 years, values are slightly lower, but the highest values are seen beneath the sulcus calcanei. As for DA, the highest values are seen beneath the posterior talar facet, cuboid facet, and inferior area of the calcaneus (6.1–10 years). In the oldest group, DA values increase also in the posterior part of the calcaneus.

### 4.3 Does Locomotor Development Affect External and Internal Calcaneal Growth?

The pre‐locomotor phase is visible in the internal morphology; unfortunately, it was not possible to apply the configuration of (semi)landmarks to the perinatal individuals (Table S1). Although, Figus, Sorrentino, et al. (2023), Figus, Stephens, et al. (2023), described a clear change in both talar morphologies at around 6 months. After this period, the external and internal morphological changes are accelerated, as the foot prepares to be loaded daily.

During the first year of life, especially during the first six postnatal months, the internal and external morphology may be characterized as unspecialized. The featureless calcaneus shape is not yet ready for its role of being weight‐bearing, and trabecular bone confirms this pre‐locomotor role, with the presence of an unspecialized architecture, that is, dense and isotropic.

The situation begins to change at the end of the first year when toddlers usually initiate the practice of unstable locomotion. This phase involves the lack of heel‐strike and toe‐off, with a plantigrade foot touching the ground. As the calcaneus grows, with an initial elongation of the body and sustentaculum tali, the internal architecture reflects the load variation with an increase in BV/TV values beneath the posterior talar facet and in the inferior‐posterior area near the metaphyseal surface. DA increases, particularly in the posterior talar facet, which is subject to compressive forces from the talus when receiving body weight. The increase in BV/TV and DA may be linked to the onset of independent locomotion.

According to Hallemans et al. (2006), children with less walking experience exhibit a wider heel, and face higher and more variable mediolateral forces when contacting the ground, probably as a direct result of their relatively greater and more varied step widths. A wider heel may help to dissipate peak forces through the foot, in addition to the presence of the heel pad and additional soft tissues. Also, loading of the calcaneus is partially due to position of the foot relative to the body, not to the leg—that is, ankle angle (Zeininger et al. 2018). Ankle angle did not differ between foot contact types, that is, flat foot‐ or initial heel‐contact, while lower vertical forces in IHC walkers suggested the presence of a heel‐to‐toe pattern or the use of the knee and hip to absorb peak forces. In adults, a slight knee yield follows heel strike, playing a pivotal role in absorbing impact forces (Hallemans et al. 2003; Zeininger et al. 2018).

Moreover, studies have shown that when on uneven terrains, also mature walkers exhibit different locomotor kinematics, with a preference for flat foot contact and higher foot clearance during leg swing (Bojsen‐Møller 1979; Gates et al. 2012; Schulz 2011). These adjustments are thought to give walkers more balance control. This way of coping with balance control is similar to that of immature walkers learning to walk. Energetic costs during walking (Voloshina et al. 2013) are greater as a result of the increased stride variability and different heights of the foot during the swing phase. The use of flatter foot contacts may affect the walking economy by reducing the total distance traveled by the body's center of mass during a step (Webber and Raichlen 2016). Holowka et al. (2022) proposed that contacting the terrain with a flatter foot, that is, increased plantar surface area, may help the walker sense the terrain through more precise “sensory feedback”. This may be true during barefoot walking. For children, the hypotheses proposed by Gates et al. (2012), that is, increasing foot contact with the ground to have more balance control, might be accompanied by the need to have greater sensory feedback. This would result in increased contact area that would allow to better accommodate balance by enabling real‐time adjustments on an uneven substrate. Increasing foot height for more effective ground clearance during the swing phase involves greater lower limb joint flexion (Schulz 2011), which consequently requires more energy expenditure due to more muscle activity.

After 3 years, BV/TV, which is highly responsive to loads, increases in the areas that are thought to be more stressed, for example, the posterior talar facet which is subject to an increase in compressive forces. BV/TV increases also in the plantar surface, which may be stressed by the plantar ligaments and linked to the development of a proper heel‐strike and toe‐off. The metaphyseal area does not show a great increase in BV/TV, which is expected as the epiphysis may play a major role in absorbing impact forces during heel strike, while there is also a protective role played by the heel fat pad. The external morphology is now more mature, as growth continues, and the surface area of the posterior talar facet and sustentaculum tali are more developed, better serving in the transmission of forces from the talus. There is also a size expansion of the plantar processes, increasing the contact area during heel strikes (Koneru and Harper 2024). DA values increase in the plantar surface and posterior area. These changes are potentially linked to the changes in gait pattern and foot function with the development of a more mature‐like pattern, that is, propulsive toe‐off and heel strike, while the longitudinal arch is still developing.

Finally, at around 6 years, the posterior and anterior talar facets continue to develop and expand, changing their orientation, as well as the more medial portion of the cuboid facet and sustentaculum tali. BV/TV values continue to increase beneath the posterior talar facet and sustentaculum tali, in particular. There is also an increase in the metaphyseal area and plantar surface. BV/TV and DA attain an adult‐like pattern, while the external surface accompanying these changes continues to expand the surface area of talar facets and expand the metaphyseal area. With the achievement of adult‐like locomotion at around 8 years, internal morphology shows an adult‐like pattern with subsequent differences likely attributable to different mobility strategies and increases in body size. Externally, the calcaneus gradually reaches a mature‐like morphology, characterized as slender and robust, and development of the sustentaculum tali and anterior talar facet are completed.

Calcaneal robustness is adapted to endure high and cyclical impact strength during walking. In particular, the calcaneal tuber, which is thought to be an adaptation for force transmission, and the lateral plantar process may offer support for accommodating ground reaction forces during heel strike (Harper, Ruff, and Sylvester 2021; Latimer and Lovejoy 1989; Prang 2015). However, heel strike is not present during the first years of life during childhood. Nevertheless, the appearance during early ontogeny of the lateral plantar process, which changes its position on the calcaneal body during ontogeny, suggests that some forces are applied constantly through growth, as is suggested by the not uncommon fracture of the apophyseal plate in children (Ogden et al. 2004). The lateral plantar process, which is large and generally plantarly positioned in humans—even though it shows some degree of variability in size and orientation—may help dissipate peak compressive forces during heel strike by contributing to increasing the contact area exposed to transmission of ground reaction forces (Boyle et al. 2018; Gill et al. 2014; Koneru and Harper 2024).

Recent research suggests that age‐related changes in BV/TV are associated with locomotor kinetics and neuromuscular maturation (Saers et al. 2022b). This work highlighted three main developmental phases that mark BV/TV trajectory: (1) a pre‐locomotor phase—for example, between the start of ossification and the onset of locomotion—that leads to a decrease in BV/TV; (2) a neuromaturation phase—for example, between the onset of locomotion and the achievement of adult‐like gait—marked by reorganization of trabecular bone and an increase in overall BV/TV; (3) a mature locomotion phase, which starts when locomotion assumes its adult‐like pattern and ends with growth completion. Our results are in line with this sequence, and some interesting conclusions may be drawn. Three periods can be observed in the developmental BV/TV trajectory in our sample, corresponding to the three phases identified by Saers et al. (2022a, 2022b). After a pre‐locomotor phase that corresponds to our perinate group and individuals younger than 6 months, the second phase (neuromaturation) started around 1 year and ended around 6 years of age. At 6 years, the achievement of an adult‐like locomotor form and maturation of the longitudinal arch takes place. In addition to these three phases, another break is observable in our sample, coinciding with the last stage of adolescence. Adolescence marks a fundamental period in the life history of an individual, in both biological and cultural worlds, and an insight into this fundamental developmental age would be necessary to test the possibility of adding a fourth phase.

## 5 Conclusion

This study investigated, for the first time, the internal and external morphology of the growing calcaneus using a whole‐bone approach. Our results match those of previous studies, confirming the influence that mechanical loading has on external and internal morphology of the calcaneus, and differs from studies that took into account less biomechanically strained bony elements. The external morphology, as seen in other elements like the talus (Figus, Stephens, et al. 2023; Hellier and Jeffery 2006), is the by‐product of genetics and mechanical forces. External morphological changes follow a determined developmental program that is further refined by mechanical forces acting on the foot while loaded in a specific manner. There are differences between populations that highlight their different backgrounds, both cultural and environmental, that act on the morphology of the calcaneus. Internal trabecular bone structure reacts differently, (re)modeling in response to mechanical loading, as locomotion progressively develops.

Some limitations of the present study may warrant further consideration. First, the archaeological sample may itself represent a potential drawback. The differences in the chronological periods here represented, as well as genetics and cultural backgrounds may have potentially influenced the patterns observed in this study. Moreover, the sample suffers from imbalanced age classes, with certain age classes being underrepresented. This lack of adequate representation of specific age cohorts may have obscured some trends in these results. Another limitation is the variation in age estimation methods used across the sample. Some methods employed are known to have lower reliability compared to others (e.g., age estimation based on long bone measurements is more susceptible to environmental factors and individual health status, such as nutritional stress or disease, than methods that rely on dental eruption). This variability in methodological accuracy could contribute to inconsistencies in the estimated age of individuals, thereby influencing the interpretation of developmental changes in the calcaneus.

Nevertheless, this work deepens current knowledge of the developmental changes in the human calcaneus linked to the development of bipedalism.

Future studies should include more individuals from different backgrounds and with known age‐at‐death information, in the absence of longitudinal studies that might shed more light on ontogeny and the forces experienced by the calcaneus. While investigating sexual dimorphism was beyond the scope of the present study, a more in‐depth focus on these potential differences during growth should also be investigated.

Another interesting point to explore further is the difference between modern and past populations. A larger sample size along with comprehensive dataset (i.e., archaeological, and cultural data) would help to evaluate how various types of footwear and even/uneven substrate affect the development of calcaneus morphology.

Further analyses are needed to better understand calcaneal development, also in combination with other foot bones since pedal elements are structurally integrated within the foot. This would help create a more nuanced and comprehensive picture of the locomotion‐related changes that foot bones collectively undergo, potentially also contributing to decrypting the complexity of the fossil record.

## Author Contributions

**Carla Figus:** conceptualization (lead), data curation (lead), formal analysis (lead), investigation (lead), validation (equal), visualization (equal), writing – original draft (lead), writing – review and editing (lead). **Kristian J. Carlson:** supervision (equal), validation (equal), visualization (equal), writing – original draft (supporting), writing – review and editing (supporting). **Eugenio Bortolini:** validation (supporting), writing – review and editing (supporting). **Jaap Saers:** data curation (supporting), writing – review and editing (supporting). **Rita Sorrentino:** data curation (supporting), writing – review and editing (supporting). **Federico Bernardini:** resources (supporting), writing – review and editing (supporting). **Antonino Vazzana:** data curation (supporting), writing – review and editing (supporting). **Igor Erjavec:** resources (supporting). **Mario Novak:** data curation (supporting), writing – review and editing (supporting). **Claudio Tuniz:** resources (supporting). **Maria Giovanna Belcastro:** resources (supporting), writing – review and editing (supporting). **Jay Stock:** writing – review and editing (supporting). **Timothy M. Ryan:** conceptualization (supporting), formal analysis (supporting), supervision (equal), validation (equal), visualization (equal), writing – original draft (supporting), writing – review and editing (supporting). **Stefano Benazzi:** conceptualization (supporting), formal analysis (supporting), funding acquisition (lead), supervision (equal), validation (equal), visualization (equal), writing – original draft (supporting), writing – review and editing (supporting). **Francesca Seghi:** writing – review and editing (supporting).

## Ethics Statement

Permits for the usage of the CT images and 3D models derived from osteological and archaeological collections have been provided by the relevant institutions (i.e., museum, research institute, university, superintendency).

## Conflicts of Interest

The authors declare no conflicts of interest.

## Supporting information


**Data S1.** Supporting Information.

## Data Availability

The data that support the findings of this study are available from the corresponding author upon reasonable request.
