# Supplementary material for: The Ontogeny of the Human Calcaneus: Insights From Morphological and Trabecular Changes During Postnatal Growth
Source: Am J Biol Anthropol. 2025 Feb 12;186(2):e70007. doi: 10.1002/ajpa.70007 (PMC11815546; doi:10.1002/ajpa.70007)
Supplement: Supplementary file 1 — Data S1. Supporting Information. [file AJPA-186-e70007-s001.docx]

**Supplementary Information**

*Table S1 – Anthropological sample*

| **Site** | **Chronological period** | **Individual** | **Age-at-death** | **Age class** |
| --- | --- | --- | --- | --- |
| **Beli Manastir** | Neolithic | G6*° | 2-3.5 y. | 1.1-3 |
|  |  | G2*° | 12-14 y. | 10.1-15 |
|  |  | G36*° | 12-13 y. | 10.1-15 |
|  |  | G4*° | 12-14 y. | 10.1-15 |
|  |  | G31* | 7.5 y. | 6.1-10 |
|  |  | G1* | 8 y. | 6.1-10 |
| **Bologna - La Certosa** | XX Century | 60-F*° | 11 m. | 0-1 |
|  |  | 58-M*° | 11 m. | 0-1 |
|  |  | 14-F*° | 1y 9m. | 1.1-3 |
|  |  | 14-M*° | 1y. 5m. | 1.1-3 |
|  |  | 7-M*° | 2 y. 9 m. | 1.1-3 |
|  |  | 8-M° | 3 y. | 1.1-3 |
|  |  | 48-F*° | 3 y. | 1.1-3 |
|  |  | 4-F*° | 5 y. | 3.1-6 |
|  |  | 5-F*° | 5y | 3.1-6 |
|  |  | 1-M*° | 5 y 8 m. | 3.1-6 |
|  |  | 6-F° | 5 y. 10 m. | 3.1-6 |
|  |  | 11-F*° | 6 y. | 3.1-6 |
|  |  | 6-M*° | 7 y. | 6.1-10 |
|  |  | 9-M° | 7 y. | 6.1-10 |
|  |  | 24-F° | 9 y. | 6.1-10 |
|  |  | 40-M*° | 9y. | 6.1-10 |
|  |  | 81-M° | 11 y. | 10.1-15 |
|  |  | 39-M* | 11 y. | 10.1-15 |
|  |  | 69-M° | 14 y. | 10.1-15 |
|  |  | 65-M° | 15 y. | 10.1-15 |
|  |  | 142-M° | 15 y. | 10.1-15 |
| **Sardinia - Cagliari** | XX Century | 17-M° | 14 y. | 10.1-15 |
|  |  | 8-M° | 15 y. | 10.1-15 |
| **Campochiaro Morrione** |  | T114° | 2 y. (± 6m.) | 1.1-3 |
|  |  | T118° | 13 y. (±1) | 10.1-15 |
|  |  | T119° | 11-12y. | 10.1-15 |
|  |  | T227° | 7 y. (±1) | 6.1-10 |
|  |  | T60° | 9 y. (±1) | 6.1-10 |
| **Faenza** | XX Century | 1-M° | 8 y. | 6.1-10 |
| **Saint John,**  **Cambridge** | 1230 – 1511 AD | 10-F354-3171---2201° | 10 y. | 6.1-10 |
|  |  | 10-F379-3248---2752° | 10 y. | 6.1-10 |
|  |  | 10-F525-2768---5077° | 8 y. | 6.1-10 |
|  |  | 10-F890-1547---284° | 10 y. | 6.1-10 |
|  |  | 10-F741-1327---2808° | 11 y. | 10.1-15 |
|  |  | 10-F390-3267---2292° | 12 y. | 10.1-15 |
| **Ostra Vetere** | 7th | T15° | 1.5 y. (1-2) | 1.1-3 |
|  |  | T19° | 3 y. (2-4) | 1.1-3 |
|  |  | T5_Ind2° | 3 y. (2-4) | 1.1-3 |
|  |  | T34° | 9 y. (8-10) | 6.1-10 |
|  |  | T24° | 9.5 y. (9-10) | 6.1-10 |
| **Parma** | XX Century | 7-F*° | 5 y. | 3.1-6 |
|  |  | 10-F*° | 13y | 10.1-16 |
| **Suasa - Domus** | 4^th^-9^th^ | SD_T18° | 1.2 y. (1-1.5) | 1.1-3 |
|  |  | SD_T44a° | 1.5 y. | 1.1-3 |
|  |  | SD_T27° | 6.5 y. (6-7) | 3.1-6 |
|  |  | SD-T42° | 11.5 y. (11-12) | 6.1-10 |
| **Suasa -** | 1st –4th | SNS_T135° | 1.5 y. | 1.1-3 |
|  |  | SNS_T134° | 2.5 y. (2-3) | 1.1-3 |
|  |  | SNS_US1127° | 7 y. (5-9) | 3.1-6 |
| **Sardinia - Sassari** | XX Century | 84-F° | 14 y. | 10.1-15 |
|  |  | 10-F° | 15 y. | 10.1-15 |
|  |  | 13-F° | 15 y. | 10.1-15 |
| **Velia** | Imperial Roman Age  1st-2nd | T322* | 36-39 w. | Perinates |
|  |  | T350* | perinate | Perinates |
|  |  | T383* | 36-39 w. | Perinates |
|  |  | T349* | perinate | Perinates |
|  |  | T352* | 38-40 w. | Perinates |
|  |  | T66* | perinate | Perinates |
|  |  | T305* | 0-3 m. | 0-1 |
|  |  | T398° | 6-8 m. | 0-1 |
|  |  | T415*° | 1-1.5 y. | 1.1-3 |
|  |  | T434° | 1-1.5 y. | 1.1-3 |
|  |  | T379*° | 2-3 y. | 1.1-3 |
|  |  | T411° | 2-3 y. | 1.1-3 |
|  |  | T342*° | 4 y. | 3.1-6 |
|  |  | T375° | 5 y. | 3.1-6 |
|  |  | T390° | 5-6 y. | 3.1-6 |
|  |  | T333*° | 6-7 y. | 6.1-10 |
|  |  | T320*° | 7-8 y. | 6.1-10 |
|  |  | T138*° | 8 y. (9-10) | 6.1-10 |
|  |  | T144° | 9-10 y. | 6.1-10 |
| Total |  | 77 individuals |  |  |

*y. = years; m. = months. The letters M and F in the Bologna codes indicate the biological sex of the individuals.*

** = trabecular analysis; ° = GM analysis*


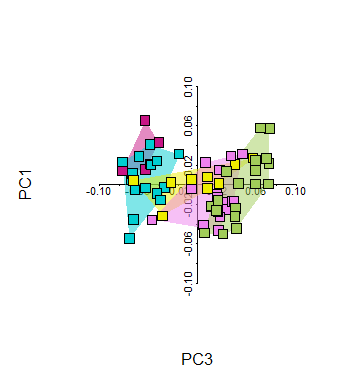

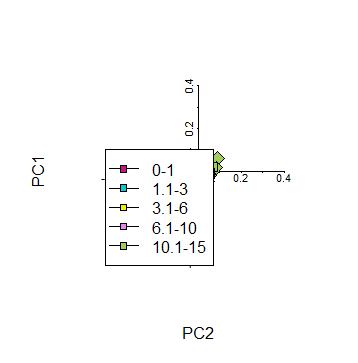


Figure S1 – Shape space plot PC1 – PC3. PC1 scores account for the 28.9% of the total variance, while PC3 scores account for the 0.9% of the total variance.


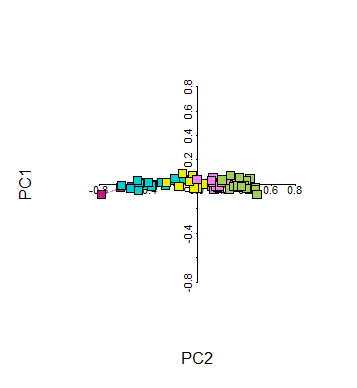

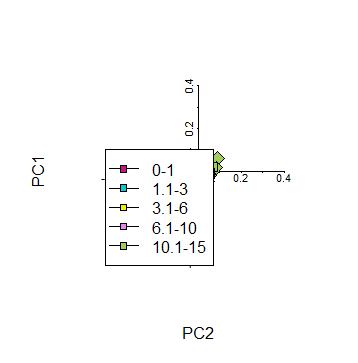


Figure S2 - Form space plot.


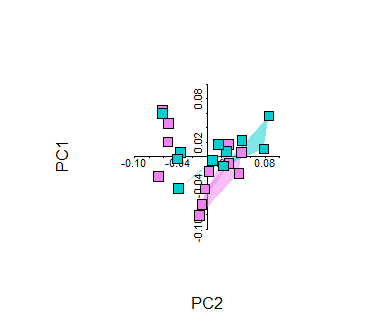


Figure S3 - PC1-2 shape space with the control sample (Bologna) pooled by sex. Males are represented in pink, and females in blue.


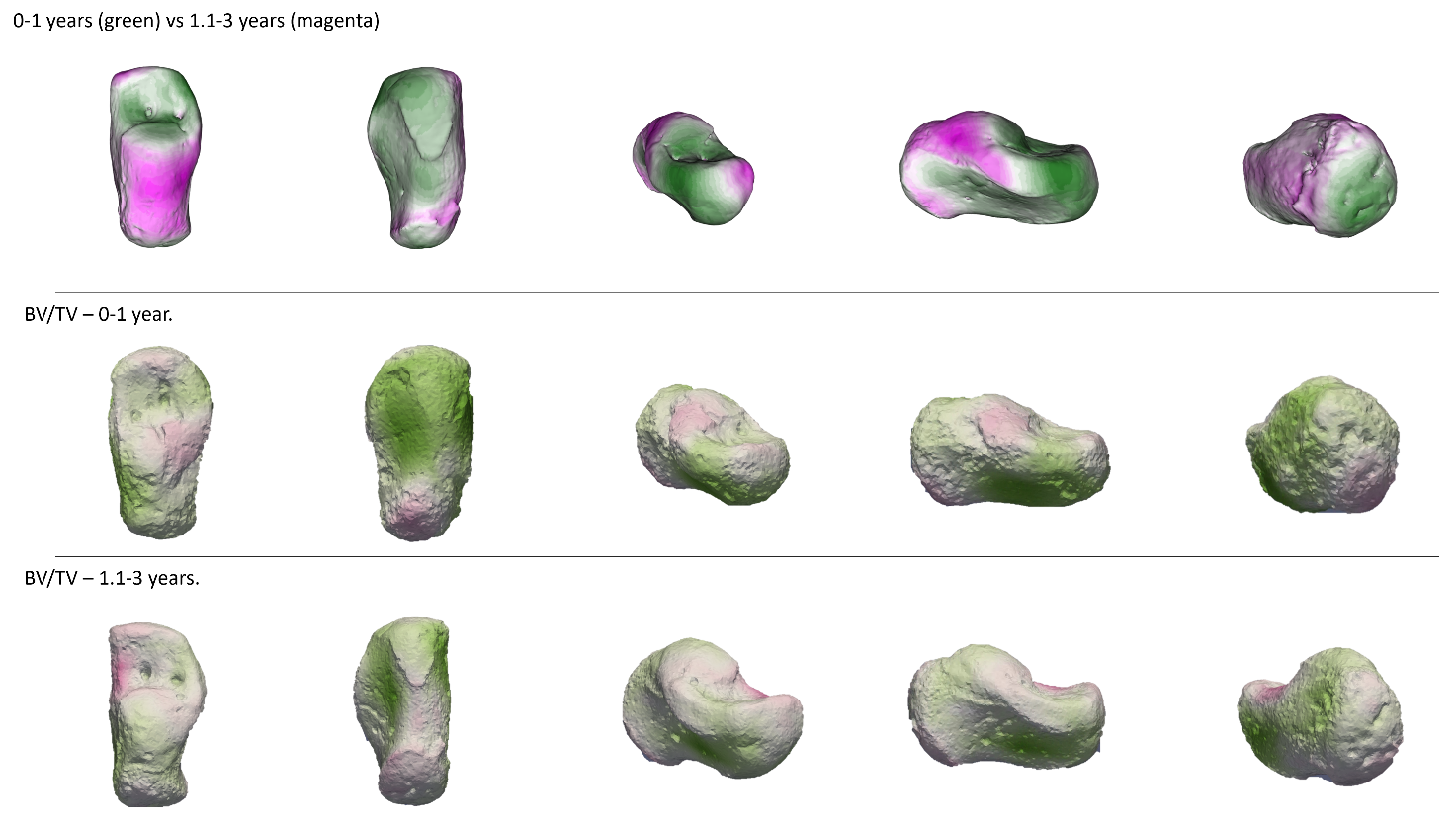


Figure S4 - Comparison between the GM heatmaps and BV/TV colormaps of classes 0-1 year and 1.1-3 years. Green represents the areas with lower BV/TV values, while magenta represents high BV/TV values. BV/TV values are higher in the posterior talar facet and metaphyseal surface. In the outer morphology, these areas develop slightly later than in the inner morphology, as pictured in Figure S2.


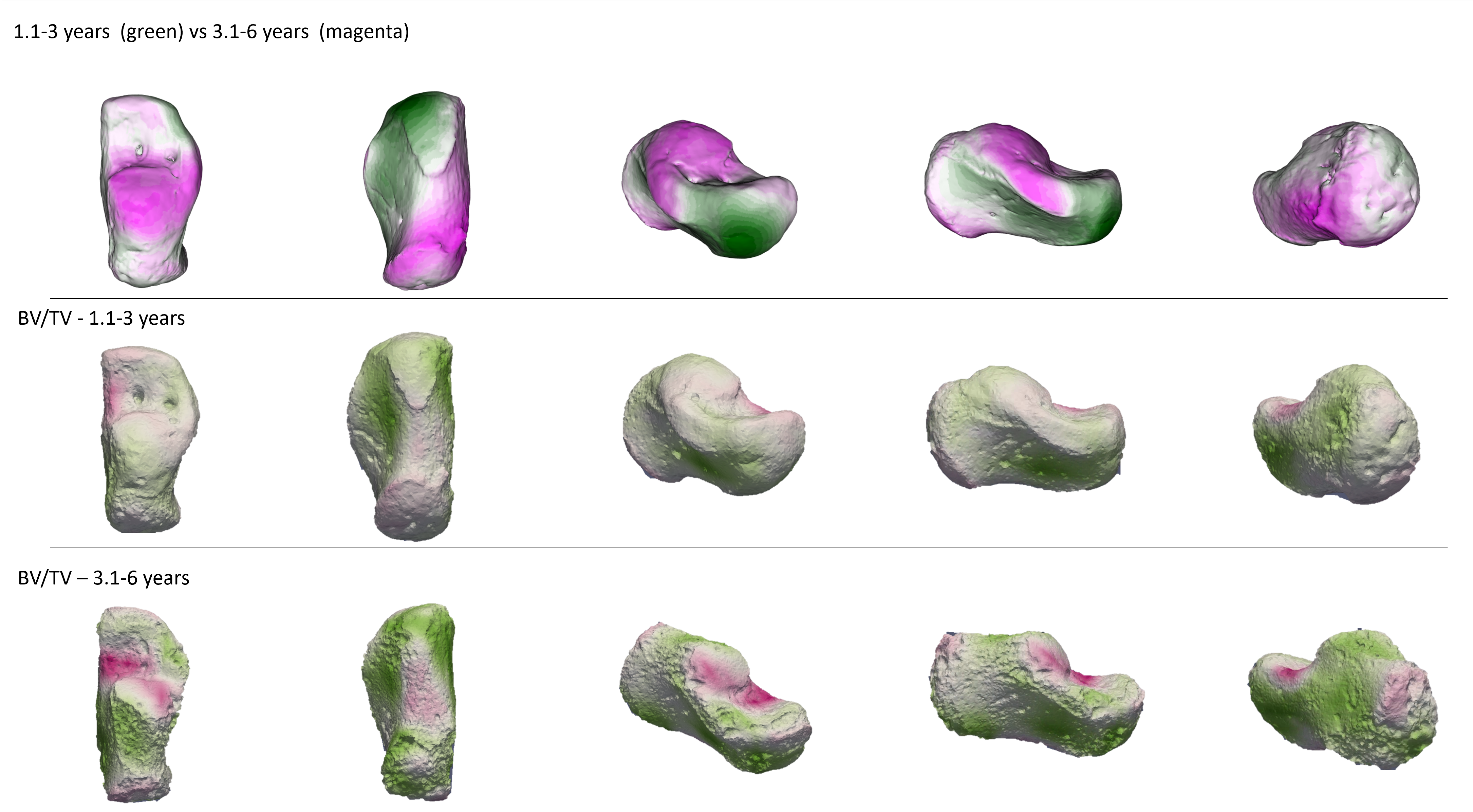


Figure S5 - Comparison between the GM heatmaps and BV/TV colormaps of classes 1.1-3 years and 3.1-10 years. Green represents the areas with lower BV/TV values, while magenta represents high BV/TV values.


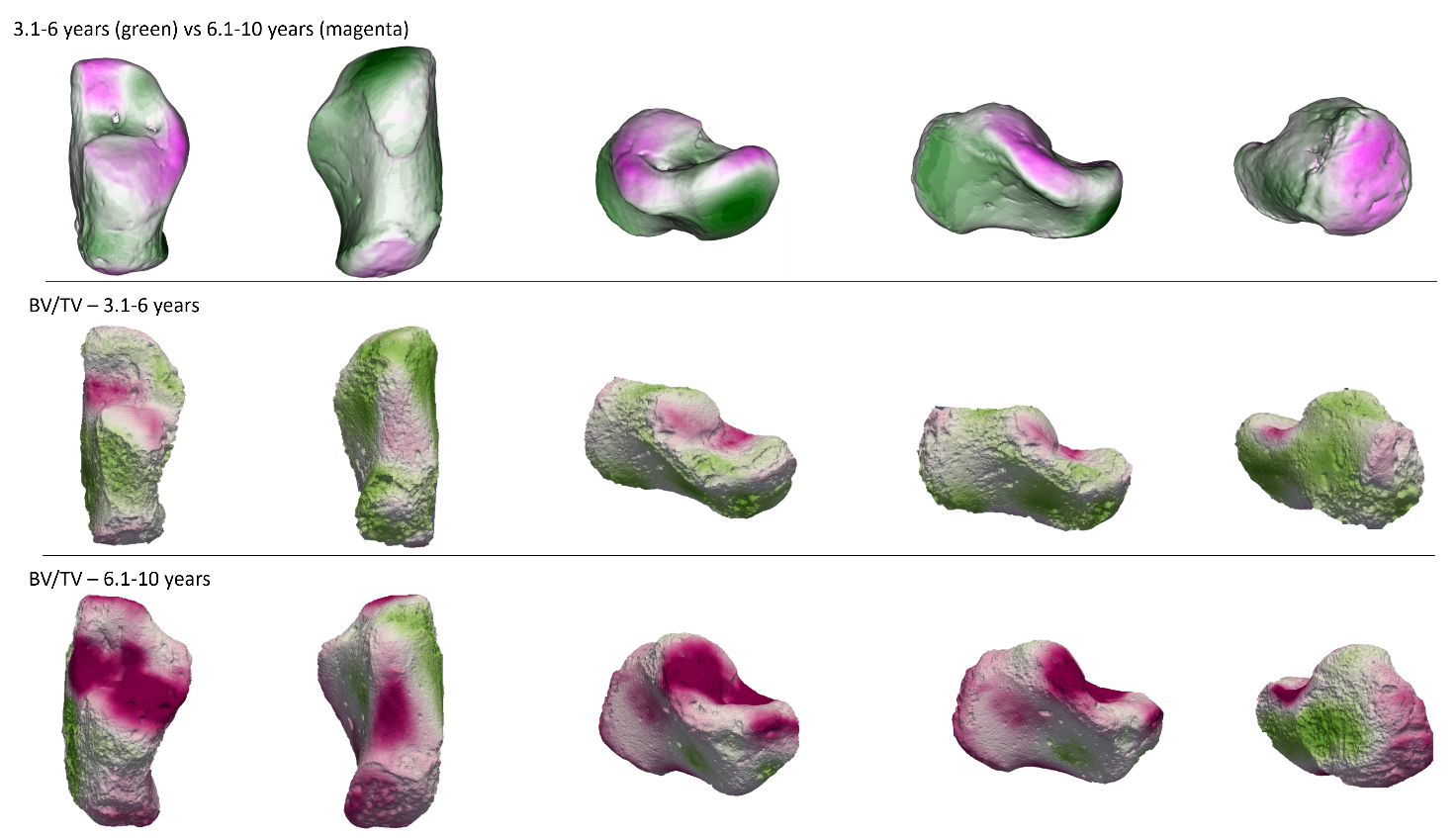


Figure S6 - Comparison between the GM heatmaps and BV/TV colormaps of classes 3.1-6 years and 6.1-10 years. Green represents the areas with lower BV/TV values, while magenta represents high BV/TV values. The development of the external and internal morphology is more similar, during this period.


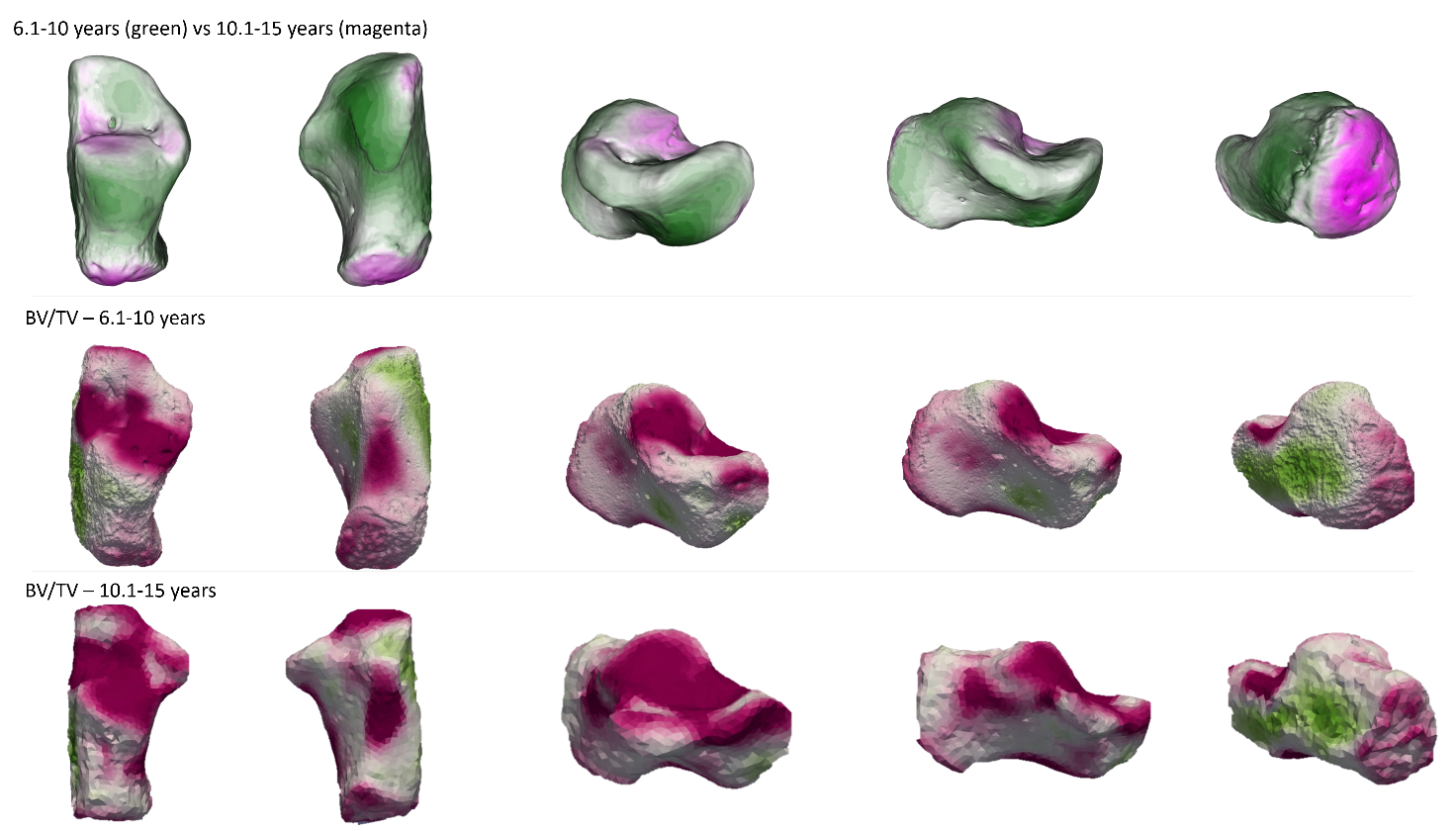


Figure S7 - Comparison between the GM heatmaps and BV/TV colormaps of classes 6.1-10 years and 10.1-15 years. Green represents the areas with lower BV/TV values, while magenta represents high BV/TV values.
